# Supplementary material for: Construction of a Myc-associated ceRNA network reveals a prognostic signature in hepatocellular carcinoma
Source: Mol Ther Nucleic Acids. 2021 May 1;24:1033–50. doi: 10.1016/j.omtn.2021.04.019 (PMC8167205; doi:10.1016/j.omtn.2021.04.019)
Supplement: Document S1. Tables S1–S4 and Figures S1–S8 [file mmc1.pdf]

OMTN, Volume *24*

Supplemental information

Construction of a Myc-associated ceRNA network reveals a prognostic signature in hepatocellular carcinoma

Dan-Dan Zhang, Yi Shi, Ji-Bin Liu, Xiao-Li Yang, Rui Xin, Hui-Min Wang, Pei-Yao Wang, Cheng-You Jia, Wen-Jie Zhang, Yu-Shui Ma, and Da Fu

Table S1. Correlation analysis between SEC14L2, SLC6A1 and biomarkers of immune cells in HCC

Description	Gene markers	SEC14L2		SLC6A1	
		Cor	P	Cor	P
CD8+ T cell	CD8A	-0.211	4.32e-05	-0.129	1.32e-02
	CD8B	-0.259	4.28e-07	-0.239	3.19e-06
T cell (general)	CD3D	-0.411	0e+00	-0.3	4.3e-09
	CD3E	-0.326	1.54e-10	-0.195	1.6e-04
	CD2	-0.346	9.7e-12	-0.203	8.81e-05
B cell	CD19	-0.24	2.86e-06	-0.121	1.96e-02
	CD79A	-0.274	8.33e-08	-0.136	8.84e-03
Monocyte	CD86	-0.282	3.78e-08	-0.235	5.04e-06
	CD115 (CSF1R)	-0.194	1.68e-04	-0.253	8.76e-07
TAM	CCL2	-0.075	1.48e-01	-0.133	1.05e-02
	CD68	-0.293	1.07e-08	-0.292	1.21e-08
	IL10	-0.23	7.39e-06	-0.156	2.51e-03
M1 Macrophage	INOS (NOS2)	0.174	7.9e-04	0.243	2.15e-06
	IRF5	-0.111	3.23e-02	0.139	7.13e-03
	COX2 (PTGS2)	-0.185	3.48e-04	-0.099	5.79e-02
M2 Macrophage	CD163	0.008	8.73e-01	-0.061	2.38e-01
	VSIG4	-0.053	3.1e-01	-0.15	3.92e-03

	MS4A4A	-0.109	3.63e-02	-0.148	4.37e-03
Neutrophils	CD66b (CEACAM8)	-0.064	2.19e-01	-0.073	1.61e-01
	CD11b (ITGAM)	-0.147	4.45e-03	-0.14	7.09e-03
	CCR7	-0.206	6.28e-05	-0.042	4.2e-01
Natural killer cell	KIR2DL1	0.049	3.46e-01	-0.004	9.37e-01
	KIR2DL3	-0.002	9.67e-01	0.003	9.59e-01
	KIR2DL4	-0.079	1.31e-01	-0.171	9.55e-04
	KIR3DL1	0.085	1.04e-01	0.039	4.59e-01
	KIR3DL2	-0.064	2.2e-01	-0.05	3.33e-01
	KIR3DL3	-0.036	4.88e-01	-0.003	9.58e-01
	KIR2DS4	0.02	6.95e-01	0.017	7.48e-01
Dendritic cell	HLA-DPB1	-0.195	1.67e-04	-0.256	6.23e-07
	HLA-DQB1	-0.209	5.37e-05	-0.23	8.06e-06
	HLA-DRA	-0.131	1.16e-02	-0.208	5.7e-05
	HLA-DPA1	-0.144	5.48e-03	-0.164	1.6e-03
	BDCA-1(CD1C)	-0.186	3.1e-04	-0.043	4.12e-01
	BDCA-4(NRP1)	-0.093	7.41e-02	-0.046	3.79e-01
	CD11c(ITGAX)	-0.274	9.59e-08	-0.156	2.6e-03
Th1	T-bet (TBX21)	-0.087	9.45e-02	-0.071	1.7e-01
	STAT4	-0.285	2.53e-08	0.006	9.05e-01
	STAT1	-0.185	3.59e-04	0.05	3.41e-01

	IFN-γ (IFNG)	-0.169	1.08e-03	-0.149	4.01e-03
	TNF-α (TNF)	-0.236	4.31e-06	-0.146	4.92e-03
Th2	GATA3	-0.302	3.04e-09	-0.145	5e-03
	STAT6	0.119	2.2e-02	0.159	2.19e-03
	STAT5A	-0.134	9.66e-03	-0.09	8.2e-02
	IL13	0.056	2.84e-01	0.189	2.56e-04
Tfh	BCL6	0.104	4.53e-02	0.081	1.19e-01
	IL21	-0.1	5.47e-02	-0.026	6.24e-01
Th17	STAT3	-0.035	5.01e-01	0.075	1.5e-01
	IL17A	-0.061	2.44e-01	0.04	4.43e-01
Treg	FOXP3	0.198	1.27e-04	0.276	6.83e-08
	CCR8	-0.201	9.49e-05	0.044	3.97e-01
	STAT5B	0.214	3.45e-05	0.423	0e+00
	TGF β (TGFB1)	-0.43	0e+00	-0.243	2.34e-06
T cell exhaustion	PD-1 (PDCD1)	-0.41	1.83e-16	-0.245	1.75e-06
	CTLA4	-0.441	4.73e-19	-0.301	3.4e-09
	LAG3	-0.209	4.97e-05	-0.159	2.08e-03
	TIM-3 (HAVCR2)	-0.345	1.19e-11	-0248	1.49e-06
	GZMB	-0.105	4.34e-02	-0.196	1.48e-04

Table S2. Correlation analysis between SEC14L2 and biomarkers of T cell (general), monocyte, TAM and T cell exhaustion in GEPIA.

Description	Gene markers	LIHC(SEC14L2)			
		Tumor		Normal	
		R	P	R	P
T cell (general)	CD3D	-0.22	***	-0.13	0.37
	CD3E	-0.16	*	-0.092	0.52
	CD2	-0.17	**	-0.11	0.44
Monocyte	CD86	-0.2	***	-0.16	0.26
	CD115	-0.14	*	-0.05	0.73
TAM	CCL2	-0.11	0.035	-0.24	0.098
	CD68	-0.14	*	-0.076	0.6
	IL10	-0.085	0.1	0.17	0.24
T cell exhaustion	PD-1	-0.15	*	0.0016	0.99
	CTLA4	-0.24	***	-0.088	0.54
	LAG3	-0.16	*	0.00031	1
	TIM-3	-0.16	*	-0.24	0.086
	GZMB	-0.15	*	-0.085	0.56

*P<0.01, ** P<0.001, *** P<0.0001.

Table S3. Univariate analysis of overall survival in LIHC patients stratified based on clinical characteristics.

Factor	Variable	N	SLC6A1		SEC14L2		Overall survival		
			median	P value	median	P value	Months (median)	95%CI	P value (Log-rank test)
Age	≧60	200	19.91	0.511	18.99	0.001	21.05	23.21-29.95	0.272
	<60	169	19.24		9.45		18.53	23.28-30.68	
Gender	male	248	18.05	0.137	15.26	0.001	19.07	23.21-29.05	0.257
	female	121	24.1		8.95		21.07	23.39-32.75	
BMI	≦18.5	21	12	0.005	8.32	0.266	19.57	14.04-43.23	0.133
	18.5-23.9	135	17.1		11.38		15.63	19.31-26.56	
	24-27.9	80	21.93		14.07		21.45	25.87-37.91	
	≧28	97	21.63		16.9		23.27	25.22-34.93	
Race	yellow	157	17.64	0.197	12.03	0.539	17.83	22.28-29.56	0.196
	white	184	21.28		13.41		22.22	24.65-32.17	
	other	28	15.7		18.66		16.65	13.53-27.80	

TNM stage	I-II	255	20.21	0.032	15.13	0.062	20.27	25.49-31.63	0
	III-IV	90	16.51		8.87		13.67	17.44-27.31	
	Unknown	24	28.31		20.48		24.52	17.05-31.18	
Diameter	< 5cm	273	20.26	0.022	15.13	0.022	19.87	24.95-30.90	0.004
	≥ 5cm	93	17.27		9.22		18.6	19.13-28.23	
Lymph-node metastasis	Negative	251	18.5	0.149	3.44	0.58	20.27	26.33-32.95	0.001
	Positive	4	27.45		4.03		20.55	5.33-34.67	
	Unknown	113	21.24		15.15		18.5	17.67-23.98	
Distant metastasis	Negative	265	18.5	0.096	12.16	0.094	20.03	25.38-31.61	0.031
	Positive	4	5.13		2.71		6.6	10.81-31.65	
	Unknown	100	21.94		16.26		18.57	18.99-26.66	
Prior malignancy	Yes	35	20.09	0.432	24.98	0.172	20.03	15.73-28.97	0.013
	No	334	19.54		12.59		19.58	24.57-29.88	

Table S4. Univariate analysis of overall survival in LIHC patients stratified based on clinical Characteristics

Factor	Variable	N	LINC02499		Overall survival		
			median	P value	Months (median)	95%CI	P value (Log-rank test)
Age	≧60	200	2.67	0.477	21.05	23.21-29.95	0.272
	<60	169	2.78		18.53	23.28-30.68	
Gender	male	248	2.88	0.683	19.07	23.21-29.05	0.257
	female	121	2.43		21.07	23.39-32.75	
BMI	≦18.5	21	2.41	0.04	19.57	14.04-43.23	0.133
	18.5-23.9	135	2.09		15.63	19.31-26.56	
	24-27.9	80	2.7		21.45	25.87-37.91	
	≧28	97	3.3		23.27	25.22-34.93	
Race	yellow	157	2.16	0.013	17.83	22.28-29.56	0.196
	white	184	3.28		22.22	24.65-32.17	
	other	28	2.6		16.65	13.53-27.80	
TNM stage	Ⅰ-Ⅱ	255	2.53	0.767	20.27	25.49-31.63	0
	Ⅲ-Ⅳ	90	3.31		13.67	17.44-27.31	
	Unknown	24	2.8		24.52	17.05-31.18	

Diameter	< 5cm	273	2.6	0.38	19.87	24.95-30.90	0.004
	≥ 5cm	93	3.32		18.6	19.13-28.23	
	Negative	251	2.42		20.27	26.33-32.95	
Lymph-node metastasis	Positive	4	3.26	0.048	20.55	5.33-34.67	0.001
	Unknown	113	3.52		18.5	17.67-23.98	
	Negative	265	2.64		20.03	25.38-31.61	
Distant metastasis	Positive	4	4.36	0.307	6.6	10.81-31.65	0.031
	Unknown	100	2.88		18.57	18.99-26.66	
Prior malignancy	Yes	35	2.1	0.297	20.03	15.73-28.97	0.013
	No	334	2.79		19.58	24.57-29.88	

Supplemental figure and legends

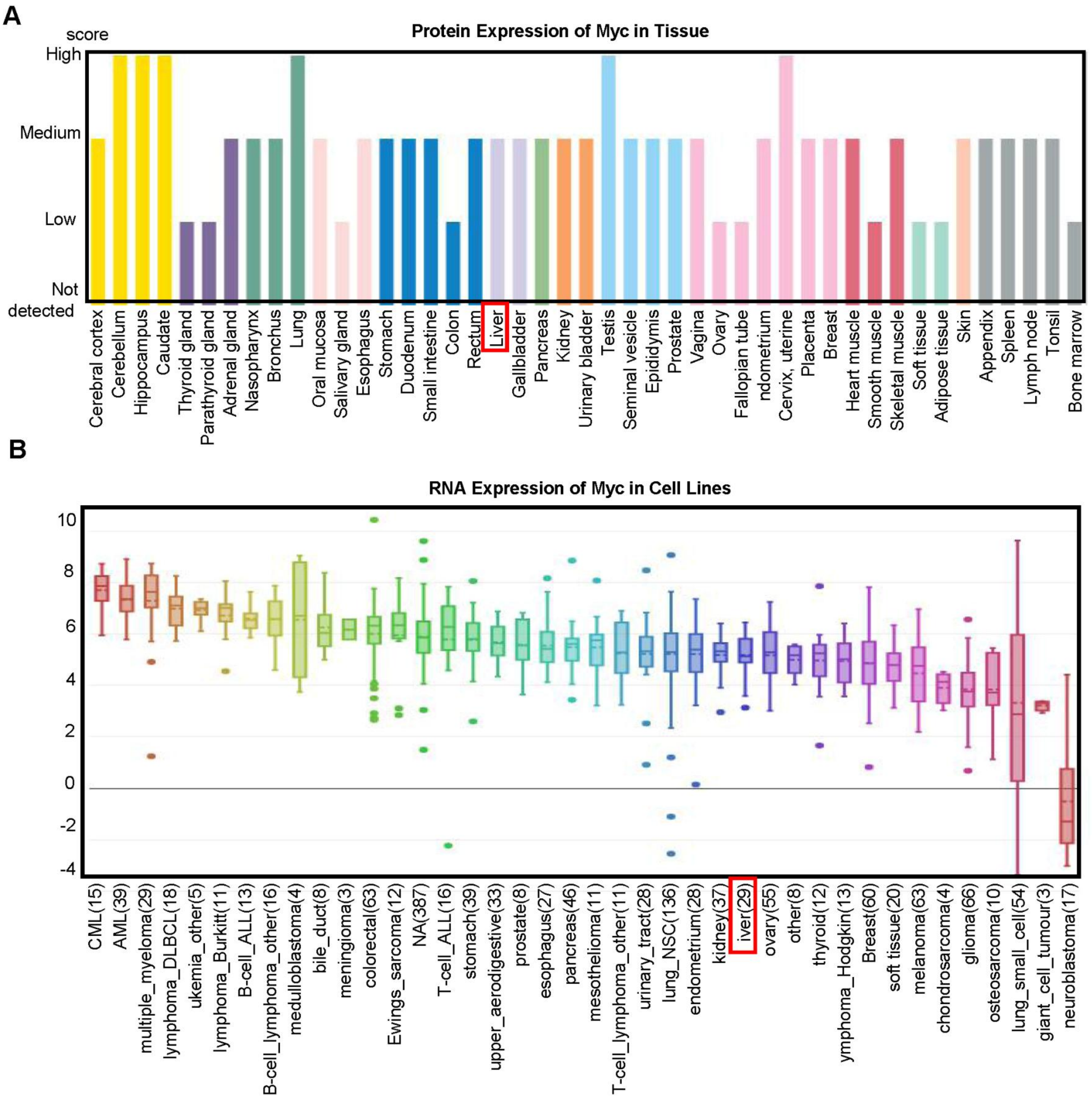

Fig. S1. Myc expression. (A) Expression of MYC in various normal organ tissues according to the HPA database. (B) Expression of Myc in cancer cell lines in CCLE.

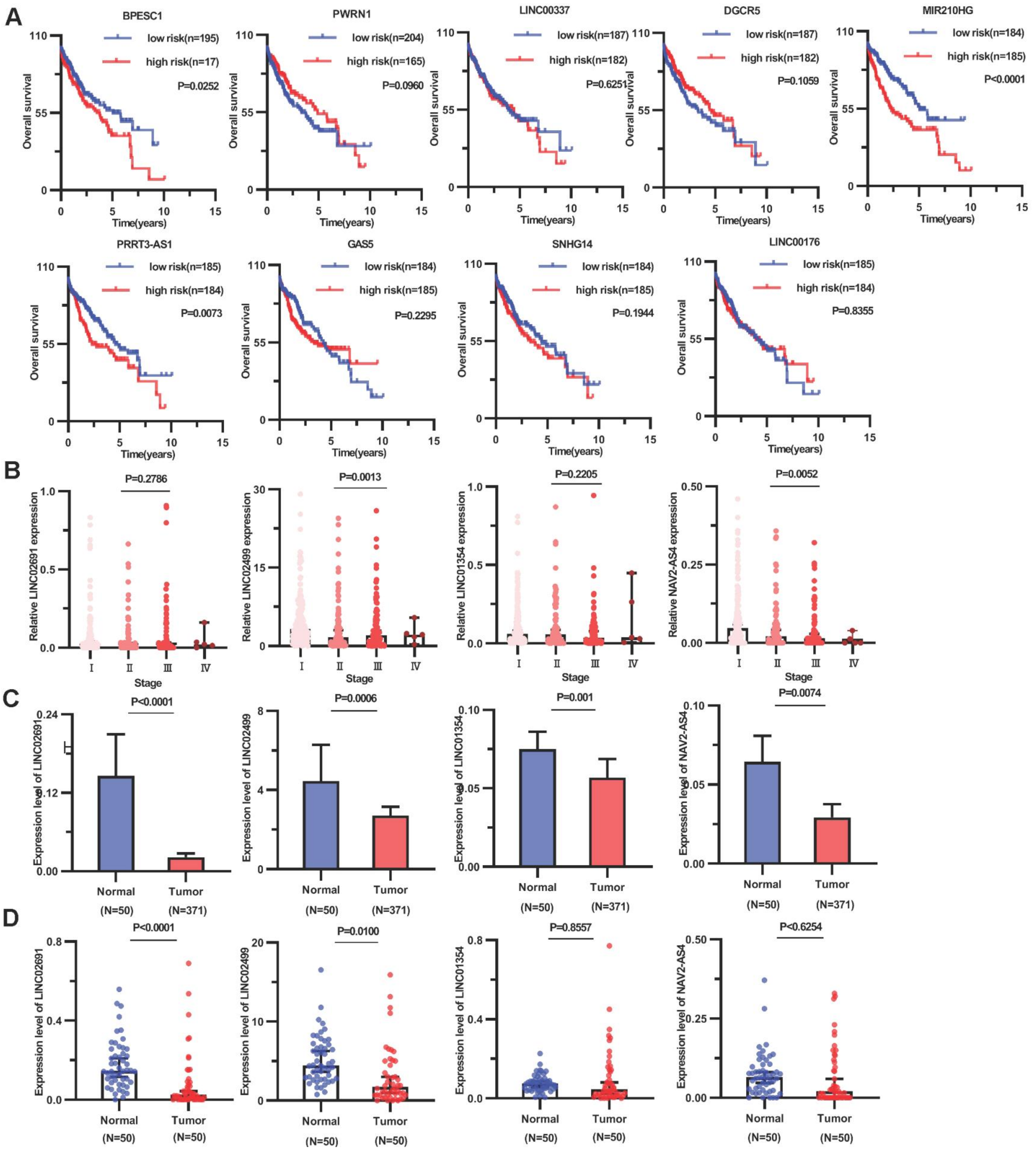

Fig. S2 Analysis of LINC02691, LINC02499, LINC01354, and NAV2-AS4. (A) Survival analysis. (B) TNM stage. (C) Differential expression in 371 HCC tissues and 50 adjacent normal tissues. (D) Validation of 50 paired cancer and paracancer tissues.

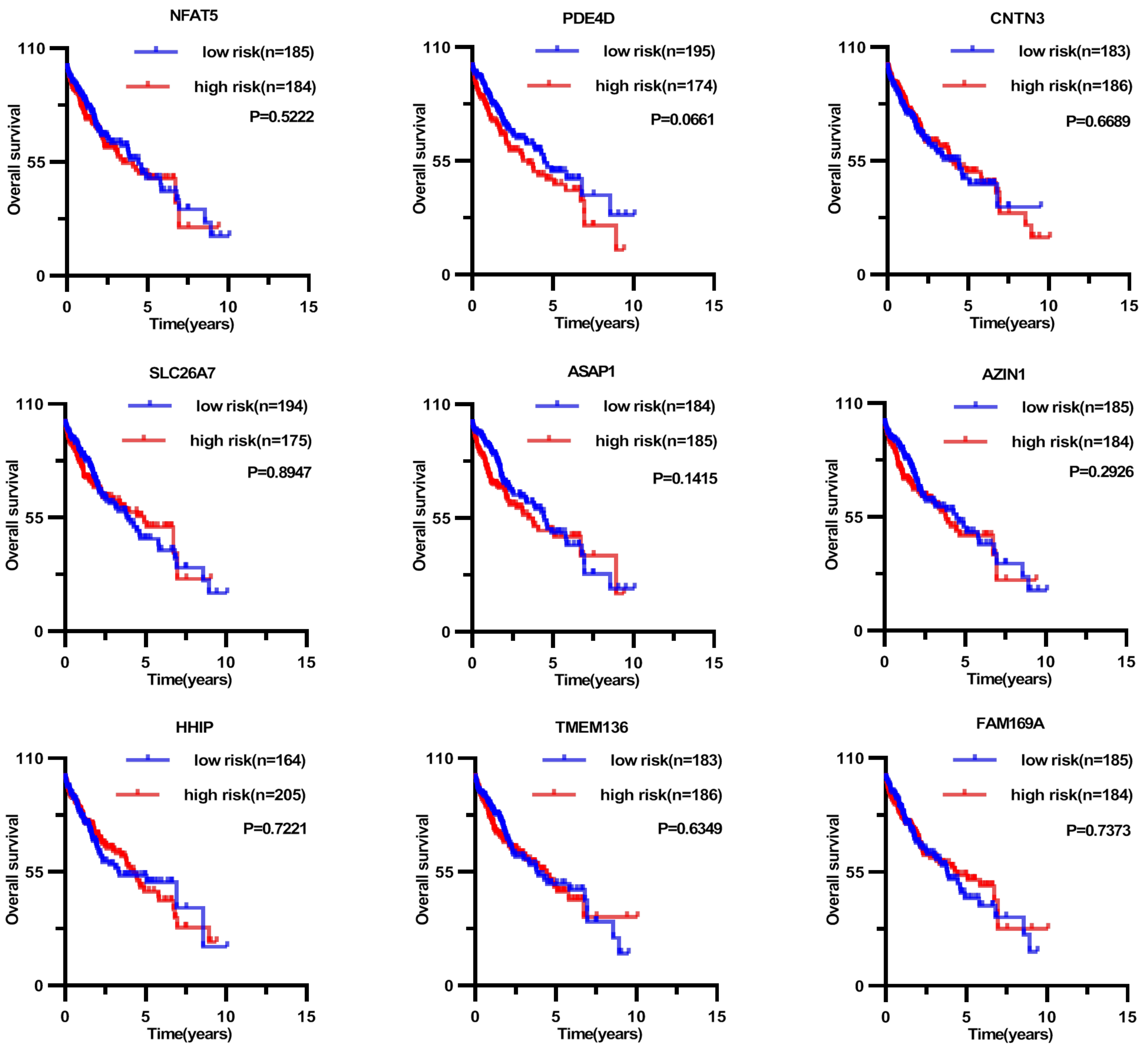

Fig. S3. Kaplan–Meier analysis of genes. Kaplan–Meier of genes that have no survival significance in the ceRNA network.

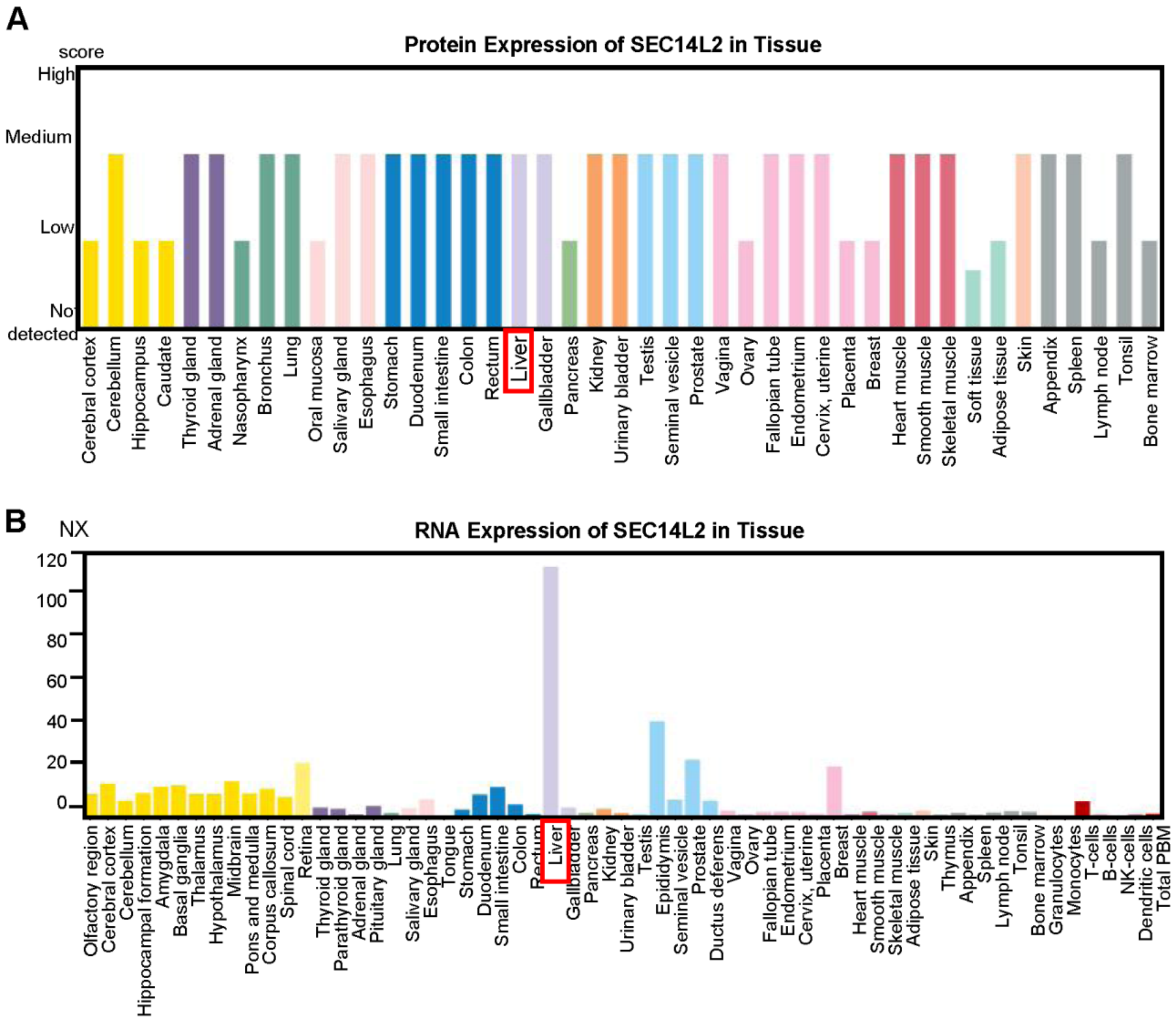

Fig. S4. The levels of SEC14L2 in various normal organ tissues. (A) Protein and (B) transcription levels of SEC14L2 in various normal organ tissues according to the HPA database.

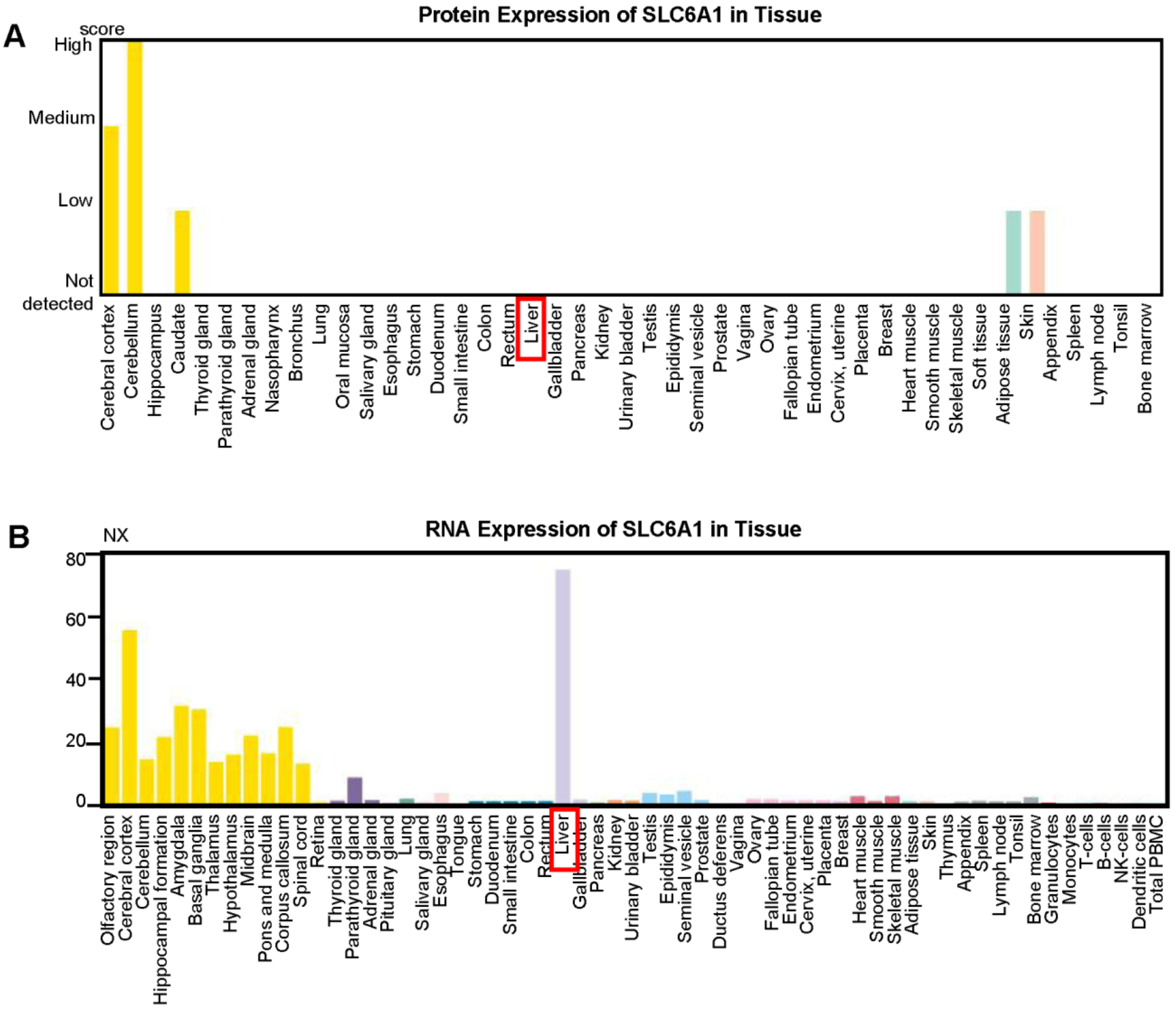

Fig. S5. The levels of SLC6A1 in various normal organ tissues. (A) Protein and (B) transcription levels of SLC6A1 in various normal organ tissues according to the HPA database.

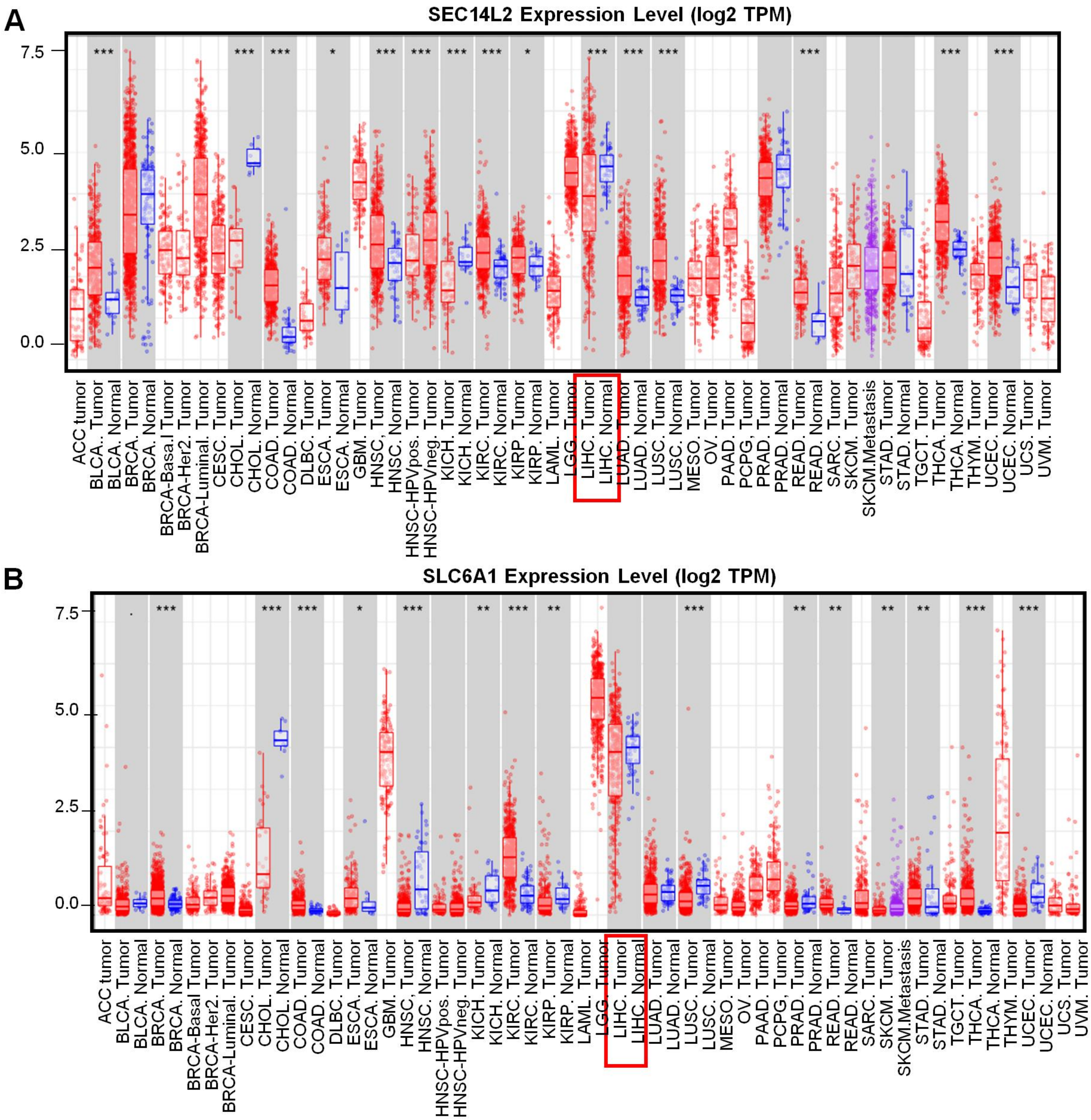

Fig. S6. The SEC14L2 and SLC6A1 expressed levels. (A) SEC14L2 and (B) SLC6A1 expression in different tumor tissues according to the TCGA database.

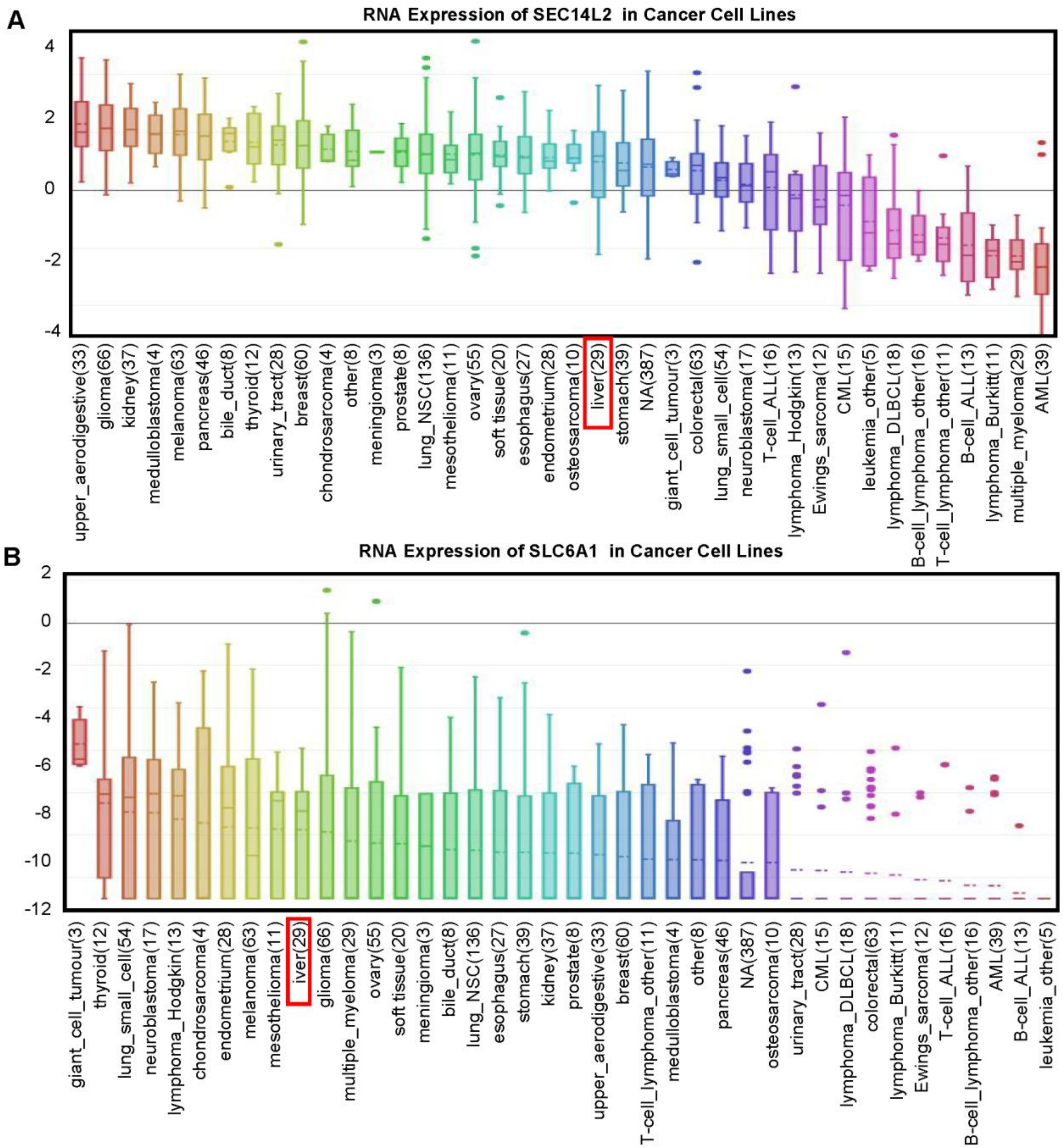

Fig. S7. SEC14L2 and SLC6A1 expression levels in cancer cell lines. The expression of (A) SEC14L2 and (B) SLC6A1 in cancer cell lines according to the Broad Institute CCLE.

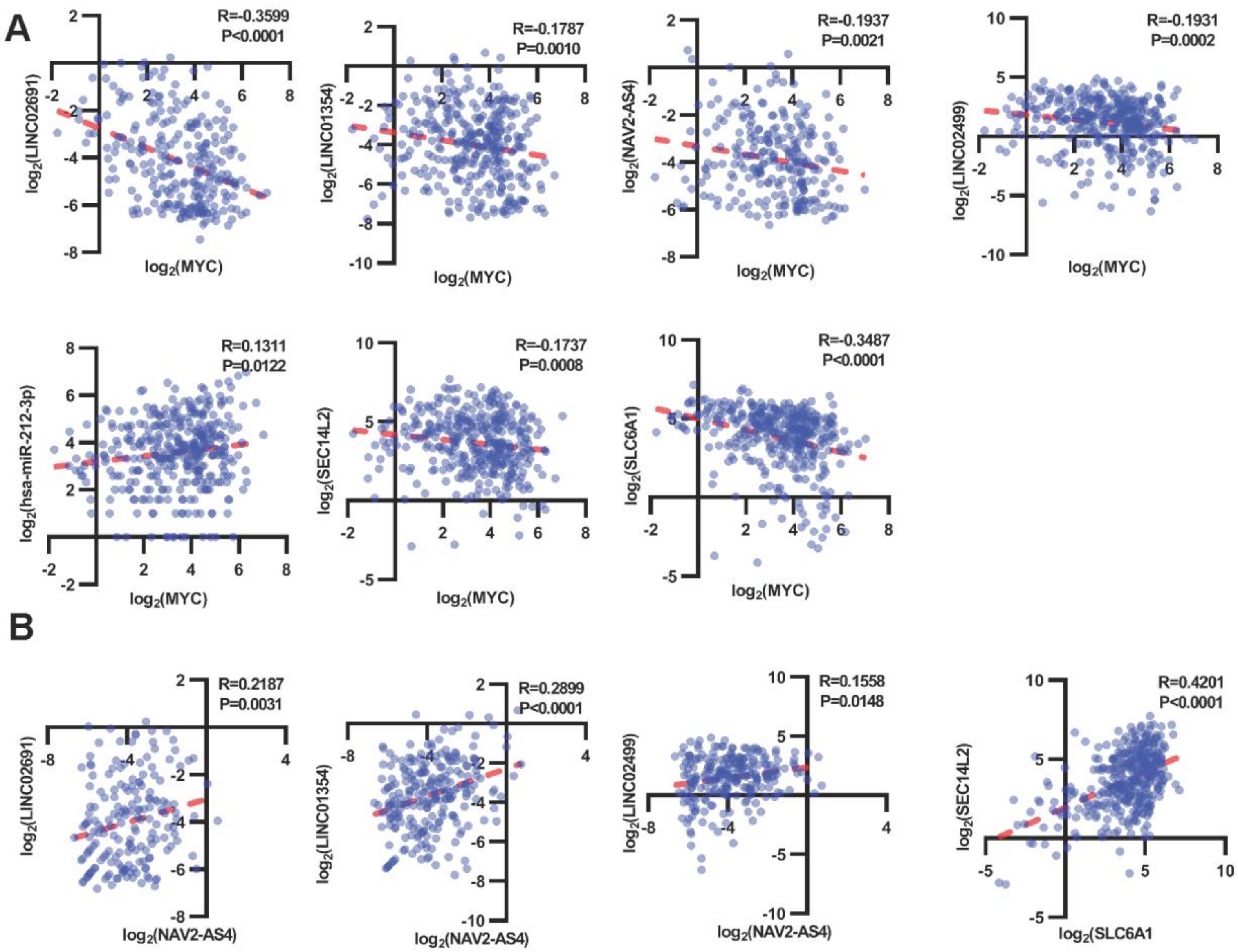

Fig. S8. Correlation among lncRNAs, miRNAs, and mRNAs. (A) Correlation between Myc and lncRNAs, miRNA, and mRNAs. (B) Remaining correlation among lncRNAs, miRNAs, and mRNAs.
