# Supplementary material for: Construction of a Myc-associated ceRNA network reveals a prognostic signature in hepatocellular carcinoma
Source: Mol Ther Nucleic Acids. 2021 May 1;24:1033–50. doi: 10.1016/j.omtn.2021.04.019 (PMC8167205; doi:10.1016/j.omtn.2021.04.019)
Supplement: Document S2. Article plus supplemental information [file mmc2.pdf]

Molecular Therapy
Nucleic Acids
Original Article

Construction of a Myc-associated ceRNA network reveals a prognostic signature in hepatocellular carcinoma

Dan-Dan Zhang,[1,2,3,7] Yi Shi,[2,7] Ji-Bin Liu,[2,7] Xiao-Li Yang,[1,7] Rui Xin,[1,7] Hui-Min Wang,[1,2] Pei-Yao Wang,[1] Cheng-You Jia,[4] Wen-Jie Zhang,[3,5] Yu-Shui Ma,[6] and Da Fu[1]

[1]Central Laboratory for Medical Research, Shanghai Tenth People's Hospital, Tongji University School of Medicine, Shanghai 200072, China; [2]Cancer Institute, Nantong Tumor Hospital, Nantong 226631, China; [3]Department of Pathology, Shihezi University School of Medicine, Shihezi, Xinjiang 832002, China; [4]Department of Nuclear Medicine, Shanghai Tenth People's Hospital, Tongji University School of Medicine, Shanghai 200072, China; [5]The Key Laboratories for Xinjiang Endemic and Ethnic Diseases, Shihezi University School of Medicine, Shihezi, Xinjiang 832002, China; [6]International Cooperation Laboratory on Signal Transduction, Eastern Hepatobiliary Surgery Hospital/Institute, National Center for Liver Cancer, the Second Military Medical University, Shanghai 200433, China

Hepatocellular carcinoma (HCC) remains an extremely lethal disease worldwide. High-throughput methods have revealed global transcriptome dysregulation; however, a comprehensive investigation of the complexity and behavioral characteristics of the competing endogenous RNA (ceRNA) network in HCC is lacking. In this study, we extracted the transcriptome (RNA) sequencing data of 371 HCC patients from The Cancer Genome Atlas platform. With the comparison of the high Myc expression (Myc^{high}) tumor and low Myc expression (Myc^{low}) tumor groups in HCC, we identified 1,125 differentially expressed (DE) mRNAs, 589 long non-coding RNAs (lncRNAs), and 93 microRNAs (miRNAs). DE RNAs predicted the interactions necessary to construct an associated Myc ceRNA network, including 19 DE lncRNAs, 5 miRNAs, and 72 mRNAs. We identified a significant signature (long intergenic non-protein-coding [LINC] RNA 2691 [LINC02691] and LINC02499) that effectively predicted overall survival and had protective effects. The target genes of microRNA (miR)-212-3p predicted to intersect with DE mRNAs included SEC14-like protein 2 (SEC14L2) and solute carrier family 6 member 1 (SLC6A1), which were strongly correlated with survival and prognosis. With the use of the lncRNA-miRNA-mRNA axis, we constructed a ceRNA network containing four lncRNAs (LINC02691, LINC02499, LINC01354, and NAV2 antisense RNA 4), one miRNA (miR-212-3p), and two mRNAs (SEC14L2 and SLC6A1). Overall, we successfully constructed a mutually regulated ceRNA network and identified potential precision-targeted therapies and prognostic biomarkers.

INTRODUCTION

Liver cancer is categorized as primary hepatic cancer and metastatic hepatic cancer.[1–3] Hepatocellular carcinoma (HCC) is the fifth-most common cancer worldwide and is a highly lethal malignancy, accounting for nearly 80% of all primary liver malignancies.[4–6] According to 2018 Global Cancer statistics,[7] there have been more than 100,000 new cases and deaths worldwide every year.

Although there have been great advancements in cancer therapy, the 5-year overall survival (OS) rate for HCC is still only 12.10%.[8–11] Surgical resection, liver transplantation, and chemotherapy are common therapies,[12–15] but they are appropriate only for patients with early-stage disease.[16,17] Therefore, it is essential to understand the mechanisms underlying the pathogenesis of HCC and identify novel biomarkers.

In the past several years, the advancement of high-throughput technologies has provided more opportunities for biomarker identification.[18–21] Non-coding RNAs (ncRNAs), which include long ncRNAs (lncRNAs) and microRNAs (miRNAs), play a role in cancer progression and are potential biomarkers and therapeutic targets.[22–25] lncRNAs are transcripts with more than 200 nucleotides, which previously were thought to have no biological function. But in fact, lncRNAs are involved in several biological processes, including cell differentiation, cancer proliferation, and metastasis.[26–29] Competing endogenous RNA (ceRNA), including lncRNA, can interact with mRNA by competitively combining different miRNAs.[30–32] miRNAs are a class of ncRNA molecules of 21–24 nucleotides that mediate negative post-transcriptional regulation.[33–35] When the miRNA arm-imbalance mechanism is broken in the cell, dysregulation of

Received 13 December 2020; accepted 28 April 2021;
https://doi.org/10.1016/j.omtn.2021.04.019.

[7]These authors contributed equally

Correspondence: Da Fu, PhD, Central Laboratory for Medical Research, Shanghai Tenth People's Hospital, Tongji University School of Medicine, 36 Yunxin Road, Shanghai 200072, China.
E-mail: fu800da900@126.com
Correspondence: Yu-Shui Ma, PhD, International Cooperation Laboratory on Signal Transduction, Eastern Hepatobiliary Surgery Hospital/Institute, National Center for Liver Cancer, the Second Military Medical University, Shanghai 200433, China.
E-mail: mayushui2006@126.com
Correspondence: Wen-Jie Zhang, MD, Department of Pathology, Shihezi University School of Medicine, Shibei 2nd Road, Shihezi, Xinjiang 832002, China.
E-mail: zhangwj82@yahoo.com

This is an open access article under the CC BY license (http://creativecommons.org/licenses/by/4.0/).

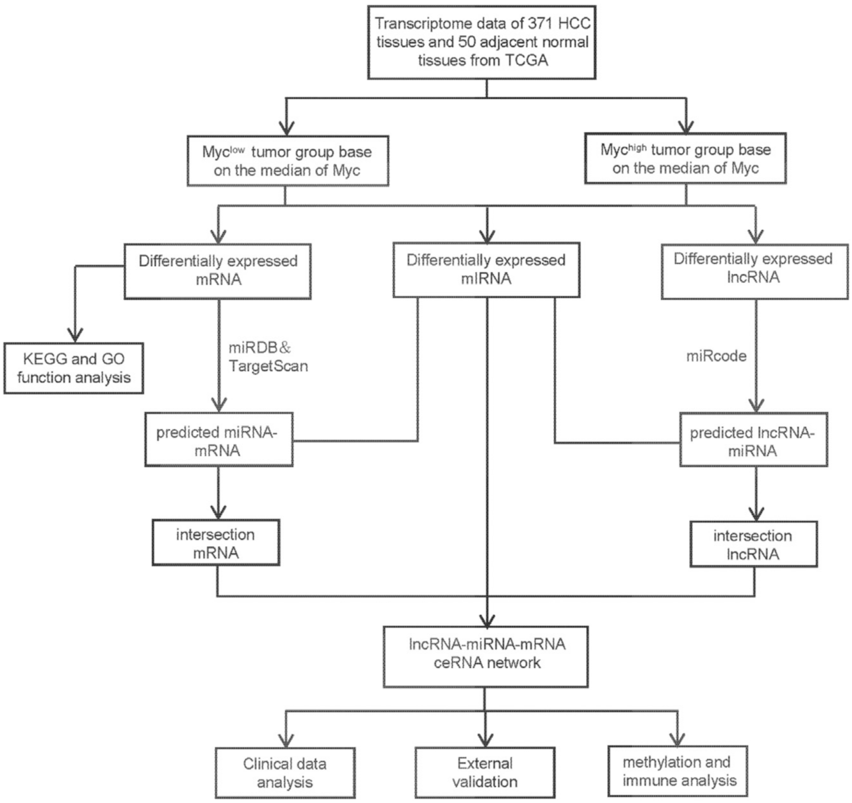

Figure 1. Research diagram of the Myc ceRNA network in HCC

downstream tumor-suppressor genes or oncogenes controlled by aberrant miRNAs occurs, leading to cancer development.[36–40] For example, lncRNA LPP antisense RNA 2 has carcinogenic effects and promotes cell proliferation and metastasis through microRNA (miR)-7-5.[41] The well-studied tumorigenic lncRNA, which is highly upregulated in liver cancer (HULC), serves as a ceRNA network through miR-372.[42] AGAP2 antisense RNA 1, a competitive lncRNA, functions as an oncogene and upregulates annexin A11 expression through miR-16-5p, promoting proliferative capacity in liver cancer.[43] These ceRNA networks provide a novel perspective and offer insight into undetected biomarkers for the early diagnosis and treatment of cancer.

The Myc gene is located on chromosome 8q24.21, which is dysregulated in most human neoplasia. This region is frequently genetically amplified in various human cancers.[44] Moreover, MYC is overexpressed in more than 50% of tumors, including HCC. In this study, we first used the median expression level of MYC to divide the 371 HCC samples into two groups for subsequent analysis: high Myc expression (Myc^{high}) tumor and low Myc expression (Myc^{low}) tumor. We used the transcriptome sequencing data of 371 lncRNAs, 371 mRNAs, and 367 miRNAs of HCC tumor tissue and adjacent normal tissue from The Cancer Genome Atlas (TCGA) platform to identify key differentially expressed (DE) RNAs, and we constructed an associated Myc ceRNA network to reveal the underlying mechanism in HCC carcinogenesis. We identified a prognostic signature (long intergenic non-protein-coding [LINC] RNA 2691 [LINC02691] and LINC02499) that effectively predicts OS and has protective effects.

We constructed a critical ceRNA network that included four lncRNAs (LINC02691, LINC 02499, LINC01354, and NAV2 antisense RNA 4 [NAV2-AS4]), one miRNA (miR-212-3p), and two mRNAs (SEC14-like protein 2 [SEC14L2] and solute carrier family 6 member 1 [SLC6A1]). This study provided a better understanding of the pathogenesis of liver cancer from the perspective of polygenic association, thus offering novel insights into targeted combination therapies.

RESULTS

Study process of transcriptome data

The study flow diagram is shown in Figure 1. We divided the transcriptome data of 371 HCC tissues into Myc^{high} tumor and Myc^{low} tumor groups based on the standard median expression of Myc. We selected DE lncRNAs, miRNAs, and mRNAs from the Myc^{high} tumor group and Myc^{low} tumor group. We considered lncRNA-miRNA to be a true interaction target by miRcode. We explored the miRDB database and TargetScan for miRNA-mRNA target prediction to construct a ceRNA network, followed by analyses of expression and survival.

Clinical analysis of Myc overexpression in HCC

To explore whether Myc affects gene expression in liver cancer, we divided liver cancer patients into Myc^{high} tumor and Myc^{low} tumor groups based on the median values of Myc in this study. The mRNA and protein levels of Myc were presented in various normal organs in the Human Protein Atlas (HPA) database (Figures 2A and S1A). The expression of Myc was high in cancer cell lines (Figure S1B). The region of Myc that is genomically altered in liver cancer was expressed through amplification and was found to be altered in 66 (18%) of 366 patients in HCC (Figures 2B and 2C). Myc expression was higher in HCC tissues ($n = 369$) compared with in normal liver tissues ($n = 160$, $p < 0.01$; Figure 2D). The expression of Myc increased as tumor invasion worsened (Figure 2E). Kaplan-Meier survival analysis showed the prognostic potential of Myc expression. These data suggested that Myc was upregulated in liver cancer samples.

DE RNAs in HCC

To better understand the relationship between DE RNAs and tumorigenesis-associated HCC, we downloaded gene-expression

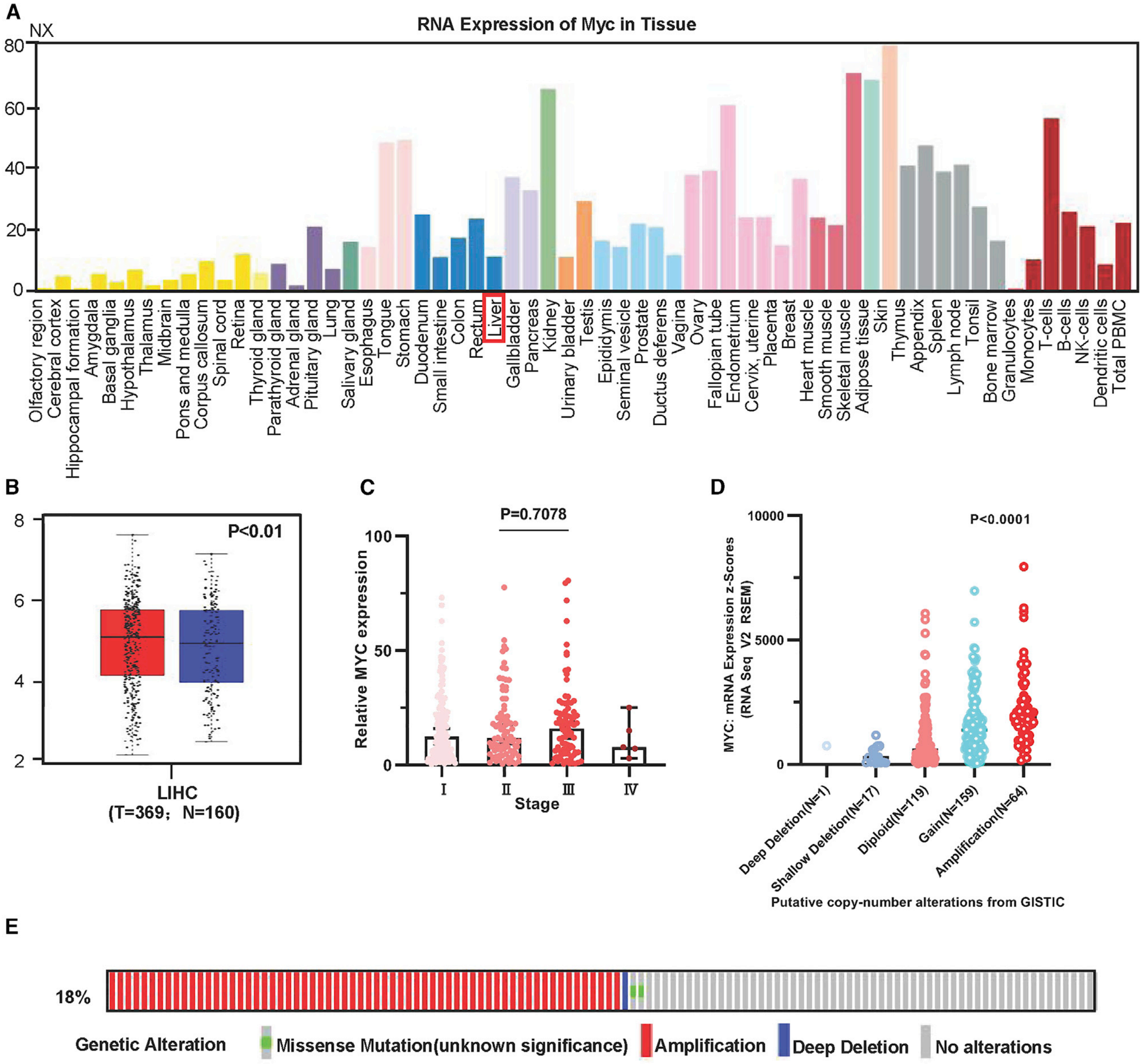

Figure 2. Functional analysis of Myc

(A) Transcription levels of Myc in various normal organs using the HPA database. (B and C) Frequency of genetic alterations associated with Myc. (D) Expression of Myc was analyzed between cancer tissues (n = 369) and normal tissues (n = 160) in HCC. (E) Distribution of Myc expression between TNM stages.

microarrays from TCGA database to search for DE RNAs. The gene-expression microarrays contained 371 samples (lncRNAs, mRNAs); 367 samples (miRNAs) were defined as Myc^{high} and Myc^{low} based on the median expression levels of Myc.[45] We analyzed the DE genes between the Myc^{high} and Myc^{low} tumor groups with the standard of $p < 0.05$ and fold change (FC) $\geq$ 1.5. In total, we found 589 lncRNAs, 93 miRNAs, and 1,125 mRNAs of DE RNAs among the groups. The upregulated DE RNAs included 222 (38%) lncRNAs, 65 (70%) miRNAs, and 696 (62%) mRNAs. The downregulated DE RNAs included 367 (62%) lncRNAs, 38 (30%) miRNAs, and 429 (38%) mRNAs. In Figure 3, the volcano plots show the distribution of DE RNAs (Figure 3A), and the heatmap describes 15 significant DE RNAs (Figure 3B).

Functional enrichment analysis of DE mRNAs

To better comprehend the mechanisms involved in HCC, we explored the function of 1,125 DE mRNAs from Gene Ontology

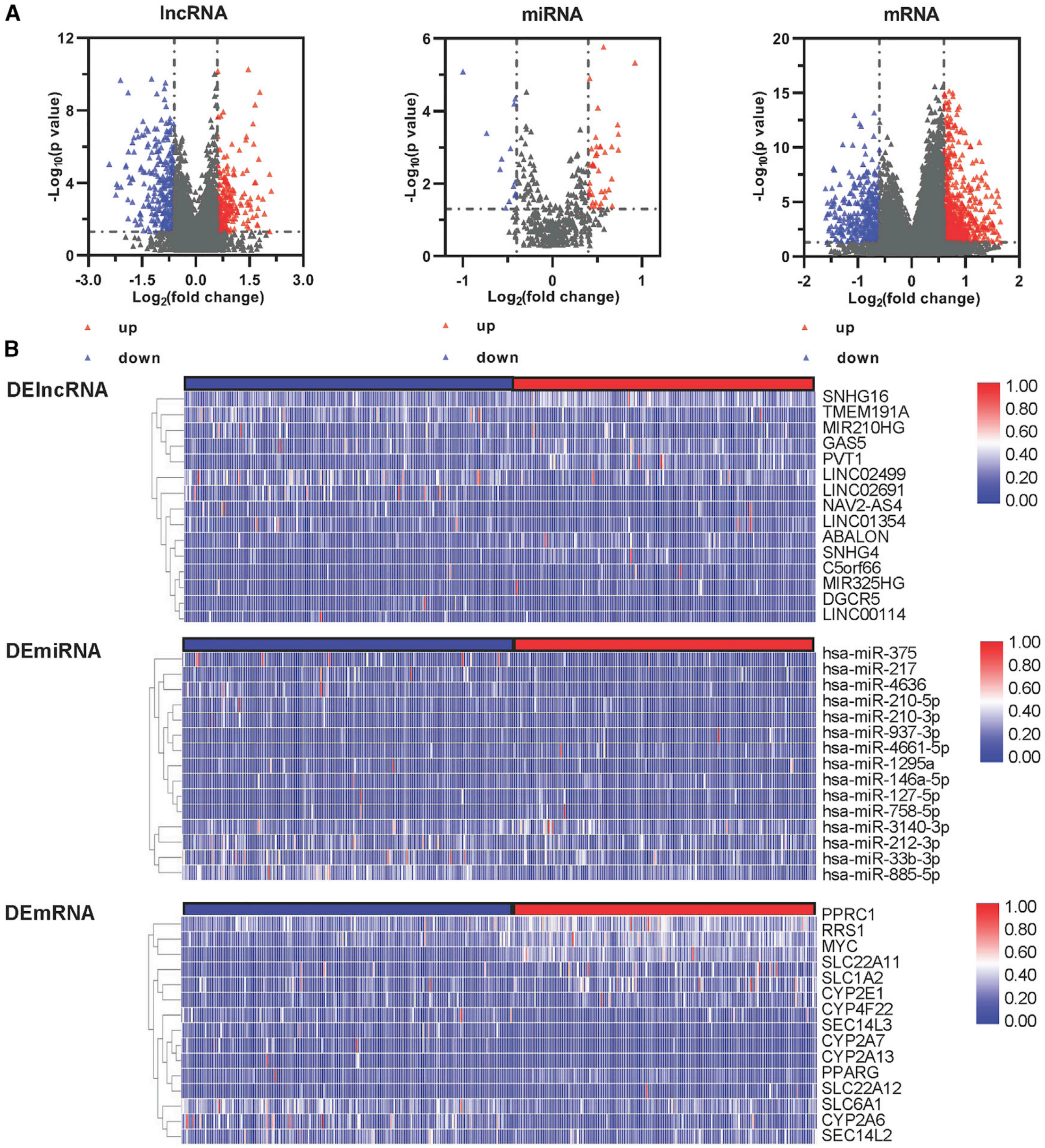

Figure 3. Identification of DE genes

(A) Volcano maps of DE lncRNAs, miRNAs, and mRNAs between two groups: Myc^{high} and Myc^{low} in HCC. (B) Heatmap of DE lncRNAs (top), miRNAs (middle), and mRNAs (bottom). Red and blue spots represent significantly upregulated and downregulated RNAs, respectively.

(GO) and Kyoto Encyclopedia of Genes and Genomes (KEGG) pathway analyses using the Metascape database (Figures 4A and 4B). The most enriched GO terms in cellular component (CC), biological process (BP), and molecular function (MF) were "side of membrane," "response to toxic substance," and "organic anion transmembrane transporter activity," respectively. We found the oxidation-reduction process, peroxisome proliferator-activated receptor (PPAR) signaling, and peroxisome pathway to participate in HCC, according to KEGG pathway enrichment analysis.

Construction of the ceRNA network and identification of hub RNAs

We used miRNA target informatic tools to identify DE RNAs in the ceRNA. The ceRNA network was constructed using Cytoscape 3.7 (Figure 5A) and included 19 lncRNAs, 5 miRNAs, and 72 mRNAs. The ceRNA network included 19 lncRNAs (11 downregulated and 8 upregulated) and 5 miRNAs (4 downregulated and 1 upregulated). In addition, 72 mRNAs (58 upregulated and 14 downregulated) and 5 miRNAs (4 downregulated and 1 upregulated) were used to construct

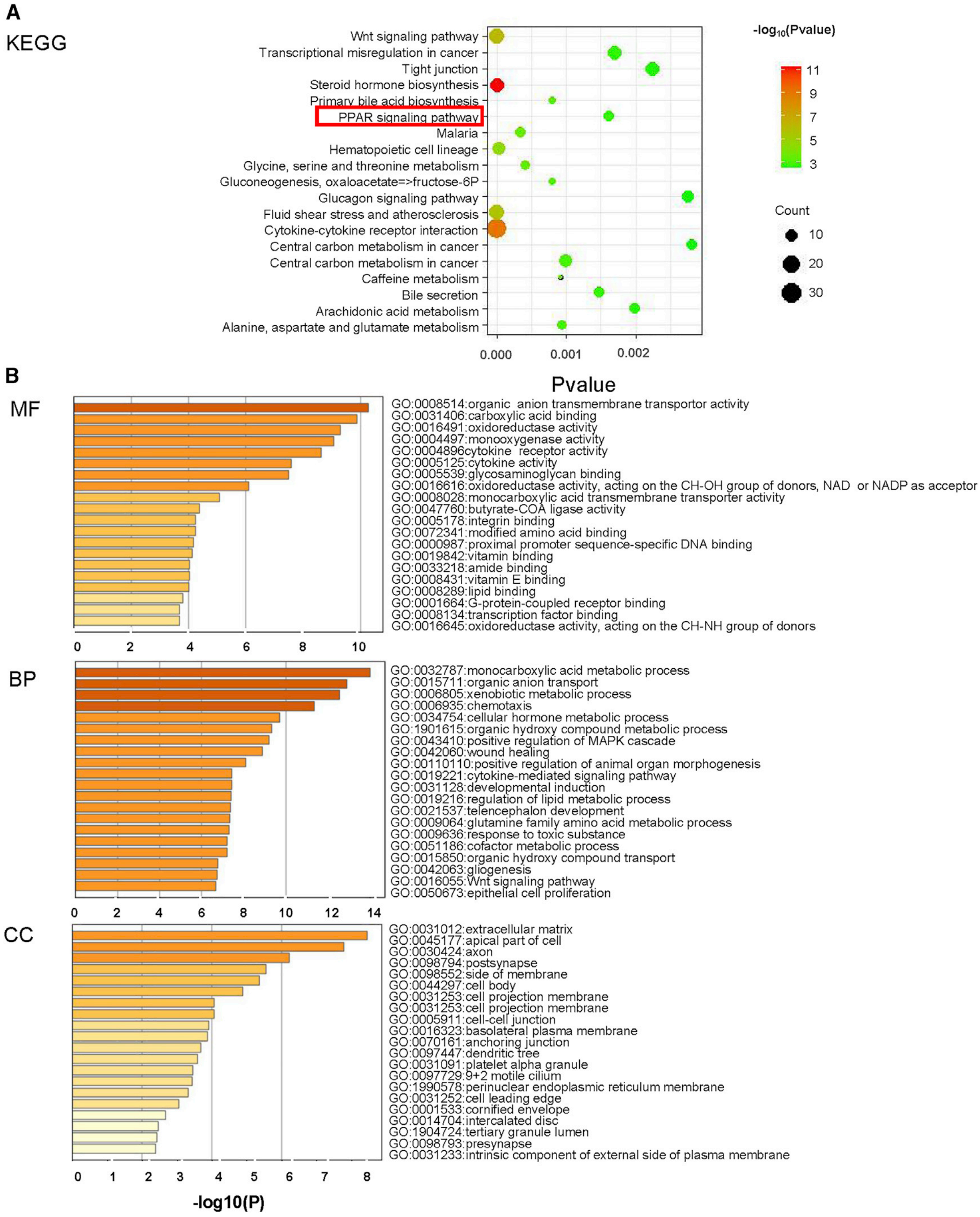

Figure 4. Functional enrichment analysis of DE mRNAs

(A) KEGG pathway of DE mRNAs. (B) MF, BP, and CC of DE mRNAs.

the ceRNA network. We employed a large ceRNA network based on node-weighting arithmetic to recognize highly interacted hub clustering (Figure 5B). The hub RNAs contained 14 lncRNAs (6 upregulated and 8 downregulated), 5 miRNAs (2 upregulated and 3 downregulated), and 11 mRNAs (9 upregulated and 2 downregulated).

Comprehensive analysis of DE lncRNAs in the ceRNA network

lncRNAs have an impact on the responses of miRNAs and mRNAs, commanding the upstream portion of the ceRNA network. The expression of lncRNAs is associated with OS in cancer patients.[46–49] Therefore, we analyzed 19 DE lncRNAs of ceRNA in this study and

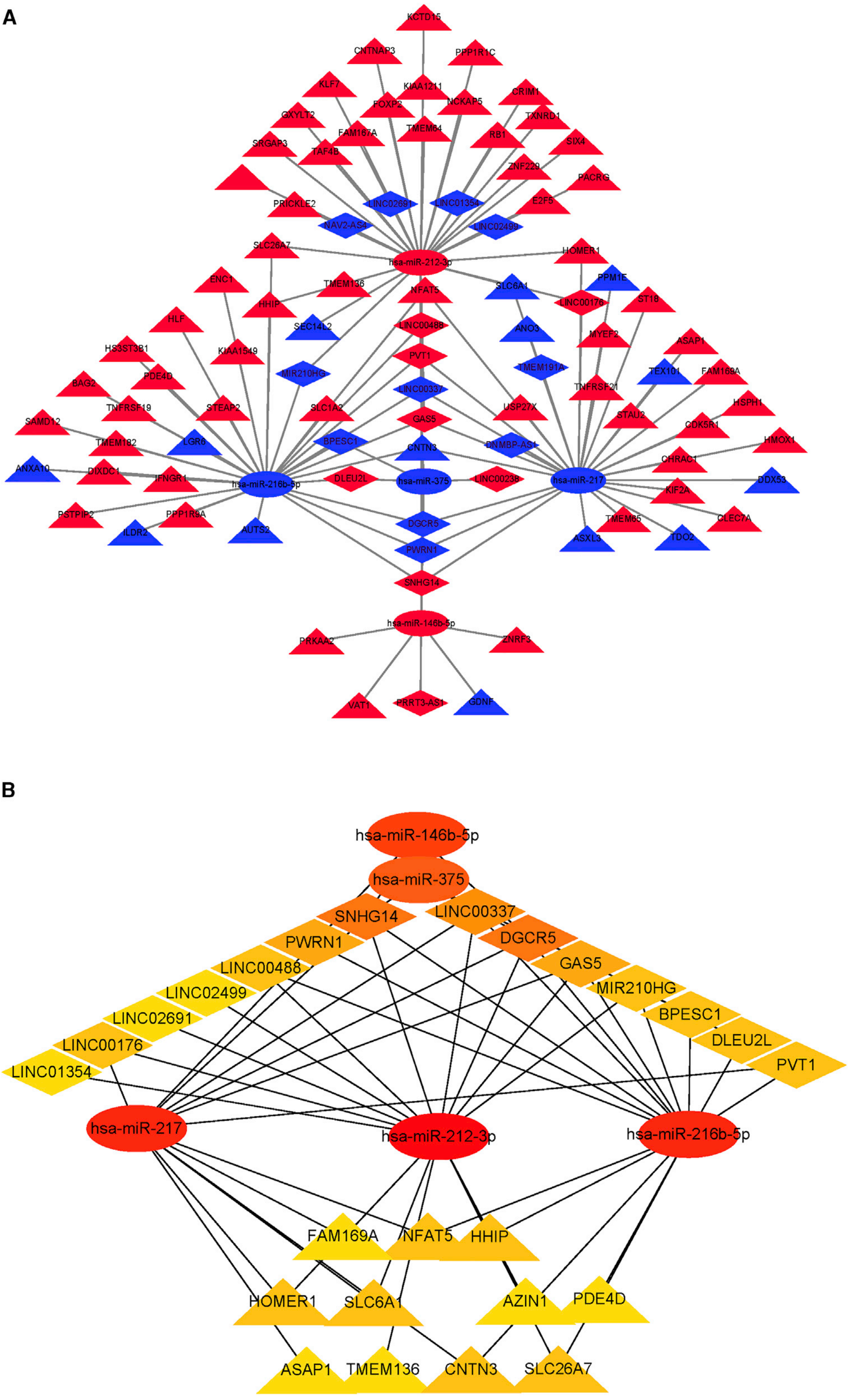

(legend on next page)

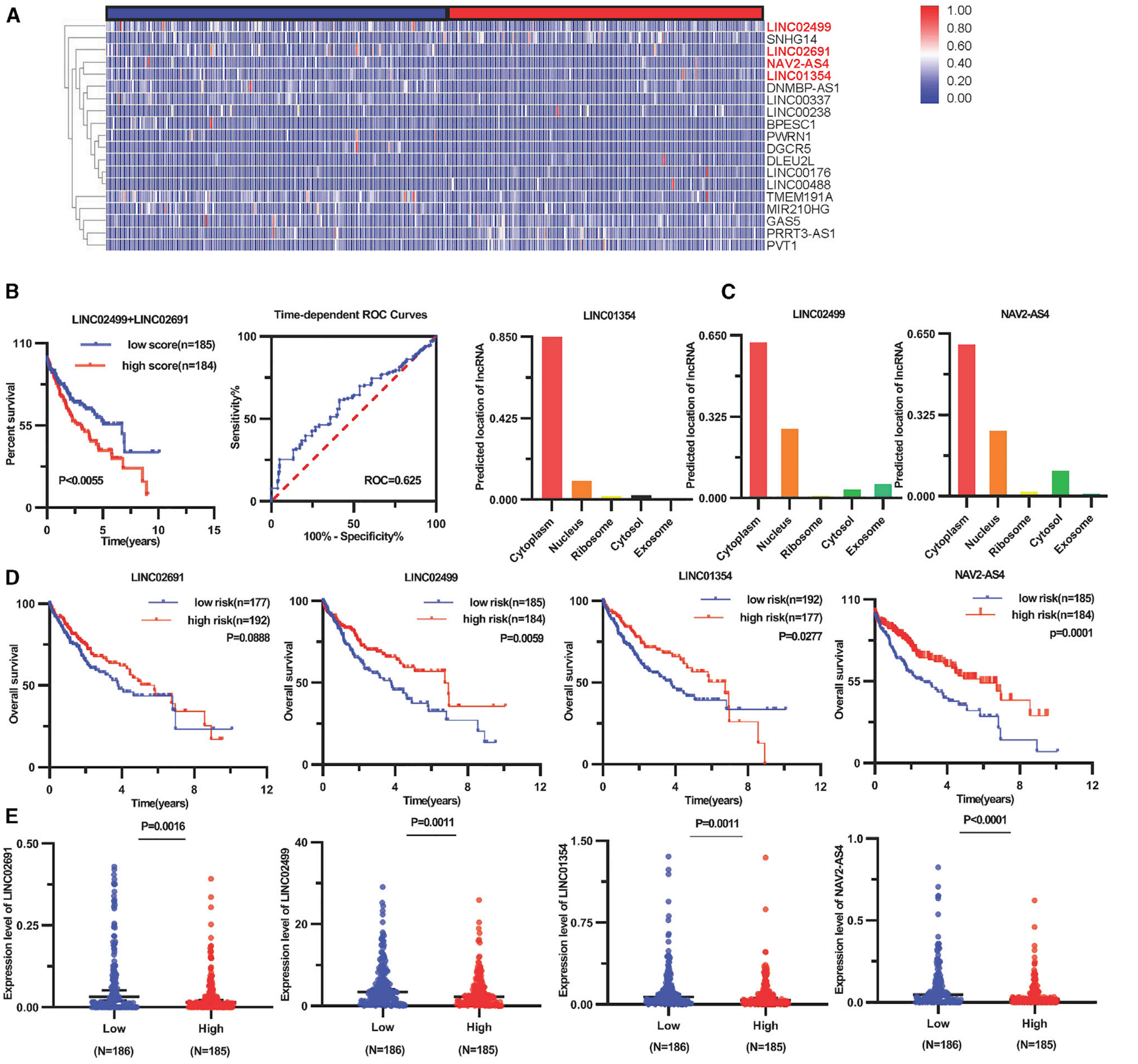

Figure 6. Comprehensive analysis of DE lncRNAs in the ceRNA network

(A) Differential expression of 19 lncRNAs in the ceRNA network by a heatmap. (B) Validation of the two lncRNA signatures by Kaplan-Meier survival and ROC curves based on risk scores. (C) Prediction of lncRNA subcellular localization; a score ranging from 0 and 1 was given. (D) Expression of LINC02691, LINC02499, LINC01354, and NAV2-AS4 was analyzed in the Myc^{high} tumor group (n = 185) and Myc^{low} tumor group (n = 186) in HCC. (E) Survival analysis.

found differential expression in the Myc^{high} tumor group (n = 185) and Myc^{low} tumor group (n = 186) using the heatmap (Figure 6A). Among the 19 DE lncRNAs, we screened potential prognosis-associated lncRNAs by univariate and multivariate Cox regression analyses. The final risk score method established that an lncRNA signal was a significant prognostic factor for HCC, as follows: prognostic index

Figure 5. Myc ceRNA network in HCC

(A) Blue rhombus represents downregulated lncRNAs, red ellipses represent upregulated miRNAs, and blue triangles represent downregulated mRNAs; red rhombus represents upregulated lncRNAs, blue ellipses represent downregulated miRNAs, and red triangles represent upregulated mRNAs. (B) The hub 30 genes in the ceRNA network.

(PI) = (−0.6621 × expression level of LINC02691) + (−0.2047 × expression level of LINC02499). Notably, the classification of these two groups using the risk score improved the distinguishing ability and predictive power regarding OS based on Kaplan-Meier ($p = 0.0055$) and time-dependent receiver operating characteristic (ROC) curve analyses (area under the curve [AUC] = 0.625). LINC02691 and LINC02499 were found to be protective factors for HCC (Figure 6B). lncRNAs of the ceRNA network play a role in the cytoplasm and are involved in the regulation of downstream genes. Hence, we used the lncLocator online tool to predict the location of LINC02691, LINC02499, LINC01354, and NAV2-AS4. We found that the subcellular locations of LINC02691 did not possess a sequences' record in LNCipedia, which was not predicted. The transcripts analysis of LINC02499, LINC01354, and NAV2-AS4 obtained from LNCipedia indicated their location in the cytoplasm using lncLocator online prediction (Figure 6C).

To identify potentially significant RNAs regarding the survival of patients with HCC, we conducted DE analysis, clinical-stage analysis, and Kaplan-Meier survival curve for each RNA in the ceRNA network. A comparison of consequences follows: 4 lncRNAs (LINC02691, LINC02499, LINC01354, and NAV2-AS4) were suppressively expressed in the Myc^{high} tumor group (n = 185) relative to the Myc^{low} tumor group (n = 186; Figure 6D); 4 lncRNAs were strongly correlated with OS ($p < 0.05$) (Figure 6E); and the remaining 15 lncRNAs were not significantly correlated with OS in HCC (Figure S2A).

We next explored the relationship of four lncRNAs according to clinical pathological stage. Following worsening tumor invasion, the expression of the four lncRNAs decreased (Figure S2B). As further verification of this finding, the difference in LINC02691, LINC02499, LINC01354, and NAV2-AS4 expression in liver cancer tissues (n = 371) was statistically significant ($p < 0.05$) compared with normal liver tissues (n = 50) (Figure S2C). The 50 paired cancer and paracancer tissues were employed to validate the results of DE lncRNAs (Figure S2D).

Functional analysis of miR-212-3p as a target

We searched for potentially significant miRNA in HCC. Five DE miRNAs (4 downregulated and 1 upregulated [miR-212-3p]) in the ceRNA network were associated with OS. We constructed a Kaplan-Meier survival curve for four miRNAs (Figure 7A). Moreover, patients with high miR-212-3p expression had a poor prognosis compared with those with low miR-212-3p expression. The results showed that miR-212-3p was a factor that promoted tumor growth. miR-212-3p was highly expressed in the Myc^{high} tumor group (n = 184) compared with the Myc^{low} tumor group (n = 18; Figure 7B). The expression of miR-212-3p increased with high tumor-node-metastasis (TNM) stage in 371 liver cancers (Figure 7C).

We performed further validation with cancer and paracancer data and compared adjacent normal tissues. We found that the expression level of miR-212-3p was significantly upregulated in 371 HCC tissues. In 50 paired HCC tissues, miR-212-3p was highly expressed in liver cancer relative to nontumor tissues (Figures 7D and 7E). In addition, through the informatics websites, we found that miR-212-3p included specific linking of the 3′ UTR region of the lncRNA gene. The 3′ UTR binding location of miR-212-3p of four lncRNAs is shown in Figure 7F. We combined miRNA and lncRNA to verify the ceRNA hypothesis. The high-risk group had high miRNA expression and low lncRNA expression, whereas the low-risk group had low miRNA expression and high lncRNA expression. We conducted survival analysis comparing the high-risk group with the low-risk group (Figure 7G).

Comprehensive analysis of DE mRNAs in the ceRNA network

To further understand the biological functions, we explored the prognostic values of the 72 DE mRNAs involved in the ceRNA network. In the ceRNA network, we found that SEC14L2 and SLC6A1 were downstream target genes of miR-212-3p. According to Kaplan-Meier survival analysis, SEC14L2 and SLC6A1 were significantly associated with prolonged prognosis ($p < 0.05$), whereas the remaining the mRNAs in the ceRNA network had no survival significance in HCC (Figure S3). SEC14L2 and SLC6A1 were overexpressed in the Myc^{low} tumor group and were associated with low-grade TNM stage (Figures 8A and 8B). Further validation showed that the expression of SEC14L2 and SLC6A in 371 liver cancers was lower than that in 50 paracancer samples; 50 samples were paired to explain the expression of the genes in cancer and paracancer tissues (Figures 8C and 8D). No genetic alterations were found in SEC14L2 and SLC6A1 in HCC; however, SEC14L2 was altered in 1 (0.3%) of 366 patients, and SLC6A1 was altered in 5 (1.4%) of 366 patients with liver cancer in the cBioPortal database. In addition, SEC14L2 and SLC6A1 had a lower expression in liver cancer (n = 10) compared with paired-normal tissue samples (n = 10) taken from the Gene Expression Omnibus (GEO) profiles (Figures 8E and 8F).

Expression of SEC14L2 and SLC6A1 in various cancers

We analyzed the transcription and protein levels of SEC14L2 and SLC6A1 in various organ tissues using the HPA database (Figures S4 and S5). To further evaluate SEC14L2 and SLC6A1 expression in human cancer, we used RNA sequencing data to examine SEC14L2 and SLC6A1 expression between tumor and normal tissues. The expression of SEC14L2 was significantly lower in some cancers, including bladder urothelial carcinoma, cholangiocarcinoma, kidney chromophobe, lung adenocarcinoma, and HCC. SLC6A1 expression was lower in most cancers, except renal cell clear carcinoma and HCC (Figure S6). The expression of SEC14L2 and SLC6A1 was low in various cancer cell lines (Figure S7).

Association of immune infiltration with SEC14L2 and SLC6A1 expression in HCC

To explore the role of SEC14L2 and SLC6A1 in immune infiltration in HCC, we evaluated the relationship between differential expression gene and immune cell infiltration by the TIMER platform. A positive correlation between SEC14L2 expression and immune cell infiltration (Cor = 0.172, p = 1.34e-03). The most positive correlation of SEC14L2 and SLC6A1 expression was with B cells (Cor = −0.271, p = 3.33e-07), $CD8^+$ T cells (Cor = −0.224, p = 3.00e-05), macrophages (Cor = −0.325, p = 7.55e-10), neutrophils (Cor = −0.224, p = 2.64e-05), and dendritic cells (Cor = −0.237, p = 1.02e-05) (Figure 8G). We

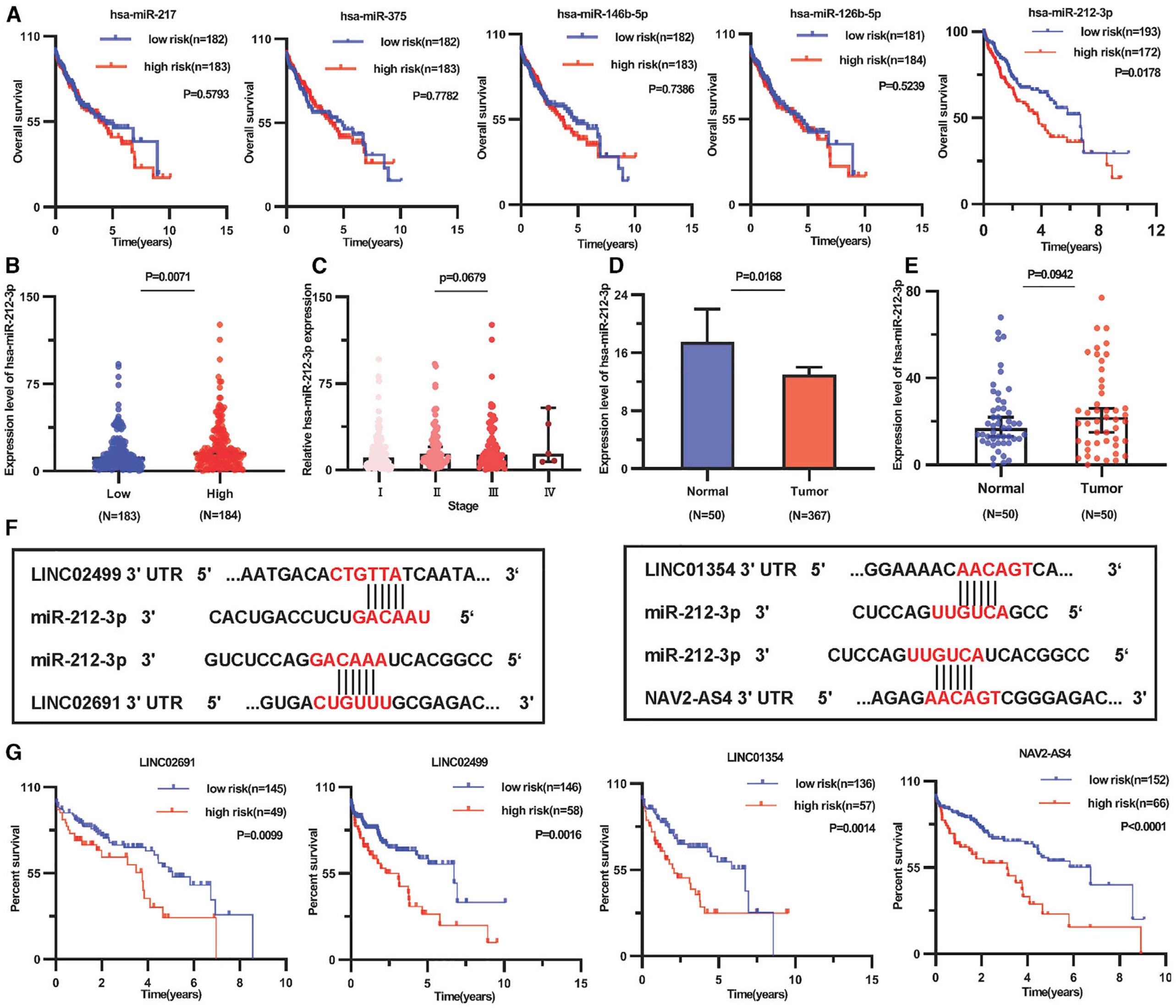

Figure 7. Expression and survival analysis of DE miRNAs

(A) Kaplan-Meier survival analysis of miR-212-3p, miR-217, miR-216b-5p, miR-375, and miR-146b-5p in 371 HCC tissues. (B) The miR-212-3p expressed in the Myc^{high} tumor group (n = 184) and Myc^{low} tumor group (n = 183). (C) The miR-212-3p expression in the TNM stage in 371 liver cancer samples. (D) Expression level of miR-212-3p in 371 HCC tissues compared with 50 adjacent normal tissues. (E) In 50 pairs of HCC tissues, miR-212-3p was highly expressed in liver cancer. (F) A schematic diagram of the miR-212-3p of 3′ UTR in four lncRNA-binding sites. (G) The high-risk group had high miRNA expression and low lncRNA expression. The low-risk group had low miRNA expression and high lncRNA expression.

researched the correlation between SEC14L2 and SLC6A1 expression and gene biomarkers of immune cells. The results were most strongly correlated with T cells and T cell exhaustion (Table S1). The correlation between SEC14L2 and gene biomarkers of immune cells in tumor and normal samples is shown in Table S2.

Correlation between methylation and SEC14L2 expression in HCC

The UALCAN database showed that SEC14L2 is highly methylated in the HCC tissue database (Figure 9A). We analyzed the relationship between SEC14L2 methylation and clinical information using the MEXPRESS database. We found significant methylation of SEC14L2 in various clinical factors, including new tumor events after initial treatment, histological type, gender, tumor stage, and OS. The methylation of SEC14L2 occurred on multiple sites, including cg03673688, cg22352499, and cg23665603 (r = 0.441, 0.447, and 0.389, respectively) (Figure 9B). We described the relationship between the methylation sites (cg03673688, cg22352499, and cg23665603) of SEC14L2 and clinical factors of patients using the MethSurv database (Figure 9C).

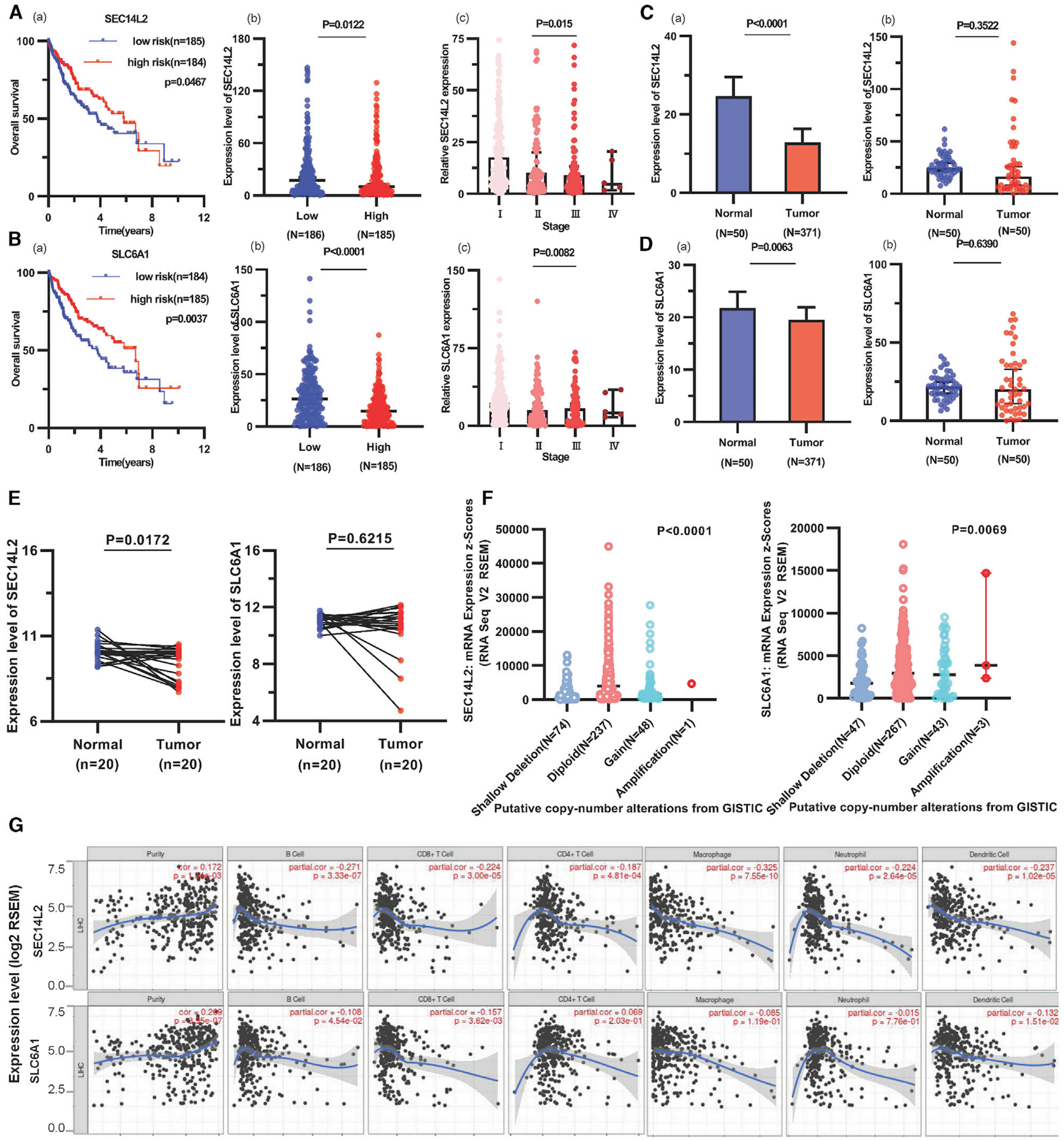

Figure 8. Analysis of SEC14L2 and SLC6A1

(A and B) Comparison of SEC14L2 and SLC6A1 expression in neoplasm development and association with prolonged prognosis. (C and D) Verification of SEC14L2 and SLC6A1 expression in normal tissues and HCC tissues in liver. (E and F) Verification of SEC14L2 and SLC6A1 in HCC relative to paired normal tissue samples from GEO profiles. (G) Immune cell infiltration of SEC14L2 and SLC6A in HCC using the TIMER database.

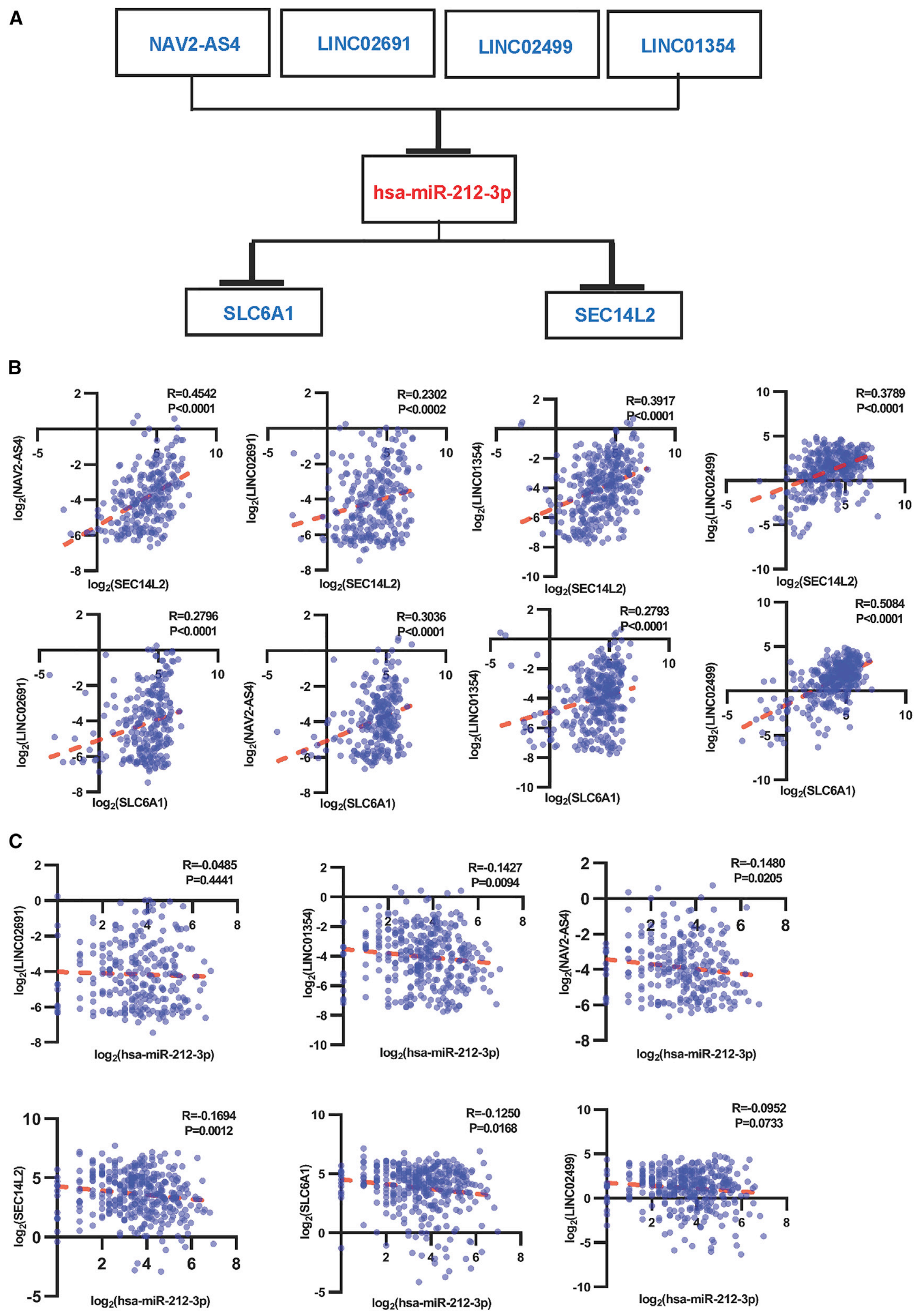

(legend on next page)

Construction of the lncRNA-miRNA-mRNA network

To verify the ceRNA mechanism in HCC, we constructed a lncRNA-miRNA-mRNA network after analysis, which included four lncRNAs, one miRNA, and two mRNAs (Figure 10A). We analyzed the correlation between Myc and these key RNAs (Figure S8A). We found that miR-212-3p is a connecting linker gene that participates in ceRNA pathways, whereas lncRNAs indirectly regulated mRNA expression by preferentially encompassing the miRNA response region. We analyzed LINC02691, LINC02499, LINC01354, and NAV2-AS4 and found that lncRNAs were positively correlated with SEC14L2 and SLC6A1 through the same miRNA, miR-212-3p (Figure 10B). Spearman correlation analysis showed that miR-212-3p was negatively regulated in lncRNAs and mRNAs of HCC (Figure 10C). The correlation among lncRNAs, miRNA, and mRNAs is shown (Figure S8B).

Relationship between key RNAs and clinical features

We analyzed the relationship between key RNAs (lncRNAs, miRNAs, and mRNAs) and clinical information, including age, gender, body mass index (BMI), race, TNM stage, distant metastasis, lymph node metastasis, diameter, and prior malignancy (Table S3). SEC14L2 and SLC6A1 had significant differences regarding BMI, tumor stage, and diameter ($p < 0.05$). TNM, diameter, lymph node metastasis, distant metastasis, and prior malignancy were significantly related to HCC prognosis ($p < 0.05$). LINC02499 was the lncRNA most significantly correlated with the clinical factors (Table S4).

DISCUSSION

HCC is the fifth-most common cancer, with the second-highest mortality rate worldwide.[50–52] The incidences of liver cancer and mortality have been increasing in the past few years, particularly in Asia. Traditional surgery is no longer the preferred treatment for HCC, so new therapeutic directions need to be explored.[17,53–55] Molecular targeted therapy is an important topic for the treatment of HCC; however, the precise molecular mechanisms remain unknown. The development of high-throughput transcriptome sequencing technologies and the ceRNA hypothesis have been proposed, in which lncRNAs and mRNAs interact with each other through shared miRNAs.[56–59] Recently, studies have been conducted to construct a ceRNA network between HCC tissues and adjacent nontumor liver tissues.[60] The molecular mechanisms of ceRNA associated with Myc remain unclear in HCC.

Myc is an oncogene that is dysregulated in >50% of tumors.[61–64] In this study, we first analyzed the large cohort of associated Myc transcriptome profiling with HCC using TCGA database. We selected DE lncRNAs, miRNAs, and mRNAs by comparing the Myc^{high} tumor tissues with Myc^{low} tumor tissues based on Myc expression, and we constructed a ceRNA network that identified 1125 DE mRNAs, 589 DE lncRNAs, and 93 DE miRNAs.

Numerous studies have reported that lncRNAs play a critical role in different cancers.[65–68] lncRNA MIR503HG inhibits cell proliferation and promotes apoptosis in triple-negative breast cancer (TNBC) cells via the miR-224-5p/HOXA9 axis.[69] lncITPF (long non-coding idiopathic pulmonary fibrosis) promotes pulmonary fibrosis by targeting hnRNP-L depending on its host gene ITGBL1.[70] lncRNA LINC00858 functions through the miR-153-3p/Rabl3 axis, which promotes cell proliferation and infiltration in HCC.[71] Therefore, we analyzed 19 DE lncRNAs in the ceRNA network by multidirectional analysis and calculated risk scores through univariate and multivariate Cox regression analyses. Our results showed that LINC02691, LINC02499, LINC01354, and NAV2-AS4 were significant in predicting OS in HCC, especially LINC02691 and LINC02499. Therefore, the prognostic signature of lncRNAs might act as a prognostic and diagnostic marker for HCC.

Among the prognostic signature lncRNAs, the oncogenic function of LINC01354 has been studied in a variety of cancers—in particular, in colorectal carcinoma, gastric carcinoma, lung carcinoma, and neck squamous cell carcinoma—in which LINC01354 accelerates the proliferation response, migration, and infiltration of cancer cells.[72–76] This study found suppression of LINC01354 expression in Myc^{high} tumor tissues compared with Myc^{low} tumor tissues. LINC02499 is highly expressed in HCC and inhibits cell-proliferation capacity, migration, and immune cell infiltration in HCC[77] and was first analyzed in the associated Myc ceRNA network. Therefore, we successfully analyzed a series of lncRNAs with functions in the associated Myc ceRNA network. lncRNAs, including LINC02691 and LINC01354, were found to be strongly negatively regulated in HCC through the miR-212-3p/SEC14L2 axis.

miRNA connects lncRNA and mRNA in various cancers, including HCC, acting as a bridge for the ceRNA network.[78–81] miR-96 reduces HCC migration, functioning as a therapeutic target in this disease.[82] miR-221-3p promotes HCC by downregulating the expression of O6-methylguanine-DNA methyltransferase.[83] In this study, we analyzed five miRNAs in the ceRNA network, and only miR-212-3p overexpression was correlated with poor OS. The ceRNA network illustrates that hsa-miR-212-3p may promote cancer cell migration and invasion.[84–87]

In this comprehensive study, we screened a ceRNA axis, including the downstream coding gene of SEC14L2 and SLC6A1. We analyzed downstream genes through GO enrichment analysis and KEGG pathway analysis. We found that the GO terms of the dysregulated mRNAs (SEC14L2 and SLC6A1) in HCC could be classified into MF, CC, and BP to further explain the pathways involved. In terms of MF, organic anion transmembrane transporter activity and vitamin E binding suggested that HCC may be a multigene-related disease. During this study, the PPAR signaling pathway was the

Figure 9. Methylation of SEC14L2

(A) Methylation of SEC14L2 in HCC tissues was described using the UALCAN database. (B) Relationship between methylation of SEC14L2 and clinical factors by the MEXPRESS database. (C) Methylation sites (cg03673688, cg22352499, and cg23665603) of SEC14L2 described by the MethSurv database.

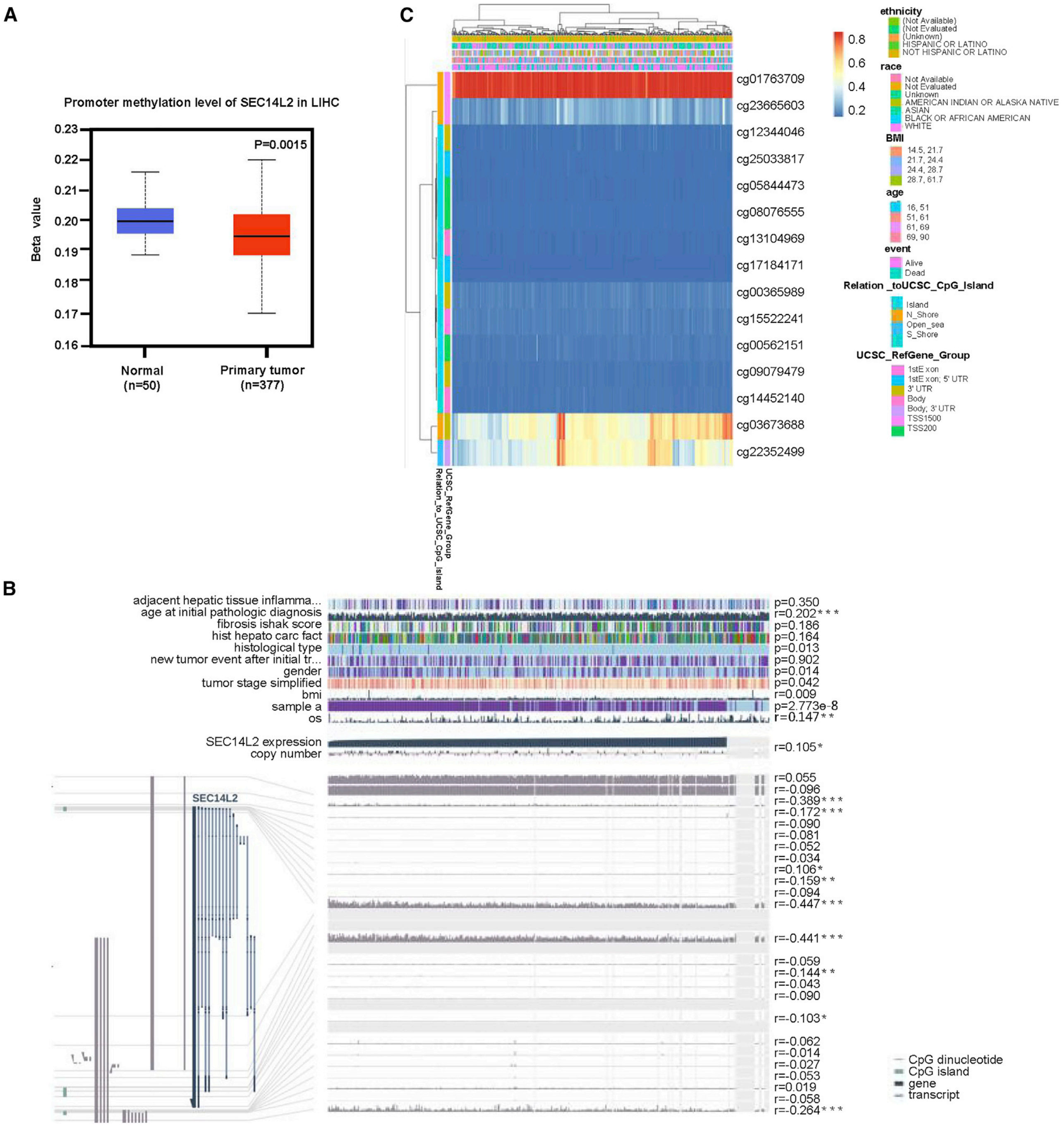

Figure 10. Correlation of linear regression analysis between DE lncRNAs and mRNAs

(A) The identified lncRNA-miRNA-mRNA axis is integrated into a circuit map. (B) Relationship of lncRNAs and mRNAs. (C) Correlation between hsa-miR-212-3p and lncRNAs and mRNAs.

most important KEGG pathway in the Metascape database, which also has been found in several other cancer types.[88–94]

SEC14L2-like phosphatidylinositol transfer proteins SEC14L3/SEC14L2 mediated Wnt/Ca^{2+} signaling by acting as GTPase proteins.[95] SLC6A1 is overexpressed in prostate cancer and is associated with drug resistance and a poor prognosis[96] but is downregulated in HCC.[97] SEC14L2 and SLC6A1 mRNAs serve as novel transcriptional targets related to Myc and suppress HCC progression and thus may be a promising target for the treatment of Myc-driven HCC. Our

results show that Kaplan-Meier survival analysis of ceRNA-correlated genes demonstrated that 19 of the 72 mRNAs had a statistically significant impact on prognosis. Many genes significantly influenced the OS of HCC patients; however, only SEC14L2 and SLC6A1 participated in establishing a complete endogenous regulation network.

This study had some limitations. Because of the lack of other HCC-associated samples, we did not perform further *in vitro* and *in vivo* experiments on clinical samples. Additionally, some exploratory experiments remain necessary to identify the functions of the unreported RNAs (lncRNAs, miRNAs, and mRNAs) in this study.

In summary, we introduced a Myc-ceRNA network from genome-wide transcriptome data by various bioinformatics analyses to provide a comprehensive analysis. This approach identified some key RNAs that were significantly associated with prognosis and that provide potential prognostic and diagnostic biomarkers for HCC.

MATERIALS AND METHODS

Data processing and analysis of DE proteins in HCC

We downloaded the transcriptome sequencing data of 421 lncRNAs and mRNAs and 417 miRNAs from 50 patients with HCC through TCGA data portal (https://portal.gdc.cancer.gov/). The 421 RNAs (417 miRNAs) were from 371 tumor tissues and 50 normal tissues. For external validation, we used 10 liver cancer samples and para-cancer samples as validation sets from GEO profiles. To study the mechanism underlying the pathogenesis of liver cancer, we defined tumor samples as Myc^{high} tumor and Myc^{low} tumor with standard median expression levels of MYC. We obtained DE mRNAs, lncRNAs, and miRNAs by comparing the Myc^{high} and Myc^{low} tumor groups, with thresholds of $p < 0.05$ and a FC of ≥ 1.5.[98]

Functional enrichment analysis of DE mRNA

Metascape (http://metascape.org)[99] is an informatics functional annotation tool that integrates many dominating databases to comprehensively analyze genes. We analyzed the enriched function of DE mRNAs using the GO and KEGG pathways in the Metascape website, with the restrictions of $p < 0.01$, a minimum count of 3, and an enrichment factor of >1.5. We visualized enriched GO pathways by Metascape and presented KEGG analyses using the “ggplot2” package of the R platform.

Construction of the ceRNA network and identification of hub RNAs

Cytoscape is a visualized software for network data, which provides users with a more picturesque biological process network.[100] We used lncRNA as a true interaction miRNA target explored by miRcode (http://www.mircode.org/) and used miRDB (http://mirdb.org/) and TargetScan (http://www.targetscan.org/) for miRNA-mRNA target gene prediction. We employed these predicted relationship pairs to construct an endogenous competitive network based on the predicted miRNA expression relationship, which was visualized by Cytoscape version 3.7.0 software. We calculated the densely connected degree of gene nodes in a ceRNA network, and we employed the top 30 genes with the highest confidence scores as hub genes.

Prediction of the subcellular location of lncRNAs

The subcellular localization of lncRNA has an impact on its function. Therefore, we predicted the sequences of the significant lncRNA biomarkers obtained from the LNCipedia (https://lncipedia.org/). Then we used the sequences of lncRNAs to predict the subcellular localization in lncLocator (http://www.csbio.sjtu.edu.cn/bioinf/lncLocator/). A score for each potential subcellular localization of lncRNA included the cytoplasm, nucleus, ribosome, cytosol, and exosome. We analyzed the final results using GraphPad Prism 8.3.

cBioPortal database analysis

cBioPortal (http://www.cbioportal.org/) is an online source application, which provides information on somatic mutations, altered copy number, and mRNA expression, used to visualize cancer genomics data.[101–104] In this study, we used cBioPortal to show SEC14L2 and SLC6A1 genetic changes in HCC.

Immune infiltrate analysis of SEC14L2 and SLC6A1

The TIMER database (http://cistrome.org/TIMER/) is a compositive resource that provides systematic analysis of the abundance of immune cells in the infiltrate of various cancers.[105] We analyzed the expression of SEC14L2 and SLC6A1 in a variety of cancers, as well the correlation between their expression levels and the abundance of the immune cells. In addition, we evaluated the correlation of SEC14L2 and SLC6A1 expression with the biomarkers of immune cells. Gene Expression Profiling Interactive Analysis (http://gepia.cancer-pku.cn/) is an open database that contains tumor and normal samples from TCGA and Genotype-Tissue Expression databases. We used this database to confirm the correlation between DE mRNA and markers of immune cells in tumor and normal samples.

Methylation analysis of SEC14L2

The UALCAN database (http://ualcan.path.uab.edu/) is a data-mining platform, in which the methylation of DE mRNA in tumors can be queried.[106–108] In this study, we used the UALCAN database to analyze SEC14L2 methylation in liver cancer tissues and paracancerous tissues. The MethSurv database (https://biit.cs.ut.ee/methsurv/) is an open website used to obtain CpG methylation data,[109] and it contains significant information about a single CpG. We screened DE mRNA using the MethSurv database and then verified the most important methylated site associated with HCC patient outcomes. MEXPRESS (https://mexpress.be/) is a data-visualization tool used to visualize TCGA expression and the relationship between methylation expression and clinical information.[110–112]

The HPA database

The HPA database (https://www.proteinatlas.org/) was initiated in 2003 to map all human proteins in cells, tissues, and organs. In this study, we assembled the protein and RNA expression levels of SEC14L2 and SLC6A1 in various cancers tissues using this database.[113–115]

Cancer Cell Line Encyclopedia (CCLE) database analysis

CCLE (https://portals.broadinstitute.org/ccle) is an open-source website, which consists of a large amount of human cancer cell lines, including genomic data, gene expression, copy number, and abundant sequencing data. We analyzed the expression of SEC14L2 and SLC6A1 in HCC cell lines using the CCLE database.

Relationship between key RNAs and clinical features

We selected key RNAs according to the previously described analyses, which are presented as the median ± standard deviation. We conducted nonparametric tests to determine whether the expression of RNAs was correlated with the following clinical features: age ($\geq$60 years versus <60 years), gender (female versus male), tumor stage (I + II versus III + IV); BMI ($\leq$18.5, 18.6–23.9, 24–27.9, and $\geq$28), race (Asian versus White), diameter ($\geq$5 cm versus <5 cm), lymph node and distant metastases (positive versus negative), and prior malignancy (yes versus no). $p < 0.05$ was used as the cutoff value.

Statistical analysis

We compared differential expression between the Myc^{high} and Myc^{low} tumor groups using the nonparametric test. The correlation and survival analysis of the relative expression of the important lncRNA-miRNA-mRNA network were processed using GraphPad Prism 8.3. We explored potential lncRNAs using Cox regression analyses in the R package version 4.0. We completed all differential expression analyses using nonparametric tests. $p < 0.05$ was considered statistically significant.

SUPPLEMENTAL INFORMATION

Supplemental information can be found online at https://doi.org/10.1016/j.omtn.2021.04.019.

ACKNOWLEDGMENTS

We would like to thank Prof. Li-Ping Gu for data analysis and critical discussion of the manuscript. This study was supported partly by grants from the National Natural Science Foundation of China (81972214, 81772932, 81472202, and 81302065); Shanghai Natural Science Foundation (20ZR1472400); Natural Science Foundation of Hunan Province of China (2020WK2020); Construction of Clinical Medical Centre for Tumor Biological Samples in Nantong (HS2016004); Jiangsu 333 Program (BRA2017205); and Wu Jieping Medical Foundation (320.6750.14326).

AUTHOR CONTRIBUTIONS

Y.-S.M., D.F., and W.-J.Z. designed the study and contributed to study materials and consumables. D.-D.Z., Y.S., J.-B.L., X.-L.Y., R.X., H.-M.W., P.-Y.W., C.-Y.J., Y.-S.M., D.F., and W.-J.Z. conducted the study. D.-D.Z., Y.S., J.-B.L., X.-L.Y., R.X., Y.-S.M., and D.F. collected data. D.-D.Z., Y.S., D.F., and Y.-S.M. performed the statistical analyses and interpreted the data. D.-D.Z., Y.-S.M., and D.F. wrote the manuscript. All authors contributed to the final version of the manuscript and approved the final manuscript.

DECLARATION OF INTERESTS

The authors declare no competing interests.

REFERENCES

1. Liu, Z., Lin, Y., Zhang, J., Zhang, Y., Li, Y., Liu, Z., Li, Q., Luo, M., Liang, R., and Ye, J. (2019). Molecular targeted and immune checkpoint therapy for advanced hepatocellular carcinoma. J. Exp. Clin. Cancer Res. *38*, 447.
2. Ma, Y.S., Huang, T., Zhong, X.M., Zhang, H.W., Cong, X.L., Xu, H., Lu, G.X., Yu, F., Xue, S.B., Lv, Z.W., and Fu, D. (2018). Proteogenomic characterization and comprehensive integrative genomic analysis of human colorectal cancer liver metastasis. Mol. Cancer *17*, 139.
3. Zhen, L., Zhao, Q., Lü, J., Deng, S., Xu, Z., Zhang, L., Zhang, Y., Fan, H., Chen, X., Liu, Z., et al. (2020). miR-301a-PTEN-AKT Signaling Induces Cardiomyocyte Proliferation and Promotes Cardiac Repair Post-MI. Mol. Ther. Nucleic Acids *22*, 251–262.
4. Zhang, X., Wang, D., Liu, B., Jin, X., Wang, X., Pan, J., Tu, W., and Shao, Y. (2020). IMP3 accelerates the progression of prostate cancer through inhibiting PTEN expression in a SMURF1-dependent way. J. Exp. Clin. Cancer Res. *39*, 190.
5. Sun, L.L., Xiao, L., Du, X.L., Hong, L., Li, C.L., Jiao, J., Li, W.D., and Li, X.Q. (2019). MiR-205 promotes endothelial progenitor cell angiogenesis and deep vein thrombosis recanalization and resolution by targeting PTEN to regulate Akt/autophagy pathway and MMP2 expression. J. Cell. Mol. Med. *23*, 8493–8504.
6. Ho, D.W., Tsui, Y.M., Sze, K.M., Chan, L.K., Cheung, T.T., Lee, E., Sham, P.C., Tsui, S.K., Lee, T.K., and Ng, I.O. (2019). Single-cell transcriptomics reveals the landscape of intra-tumoral heterogeneity and stemness-related subpopulations in liver cancer. Cancer Lett. *459*, 176–185.
7. Bray, F., Ferlay, J., Soerjomataram, I., Siegel, R.L., Torre, L.A., and Jemal, A. (2018). Global cancer statistics 2018: GLOBOCAN estimates of incidence and mortality worldwide for 36 cancers in 185 countries. CA Cancer J. Clin. *68*, 394–424.
8. Yu, F., Chen, B., Dong, P., and Zheng, J. (2020). HOTAIR Epigenetically Modulates PTEN Expression via MicroRNA-29b: A Novel Mechanism in Regulation of Liver Fibrosis. Mol. Ther. *28*, 2703.
9. Hutchings, C., Phillips, J.A., and Djamgoz, M.B.A. (2020). Nerve input to tumours: Pathophysiological consequences of a dynamic relationship. Biochim. Biophys. Acta Rev. Cancer *1874*, 188411.
10. Siemers, N.O., Holloway, J.L., Chang, H., Chasalow, S.D., Ross-MacDonald, P.B., Voliva, C.F., and Szustakowski, J.D. (2017). Genome-wide association analysis identifies genetic correlates of immune infiltrates in solid tumors. PLoS ONE *12*, e0179726.
11. Hu, J., Dong, Y., Ding, L., Dong, Y., Wu, Z., Wang, W., Shen, M., and Duan, Y. (2019). Local delivery of arsenic trioxide nanoparticles for hepatocellular carcinoma treatment. Signal Transduct. Target. Ther. *4*, 28.
12. Zhao, Y., Zhang, Y.N., Wang, K.T., and Chen, L. (2020). Lenvatinib for hepatocellular carcinoma: From preclinical mechanisms to anti-cancer therapy. Biochim. Biophys. Acta Rev. Cancer *1874*, 188391.
13. Jasirwan, C.O.M., Hasan, I., Sulaiman, A.S., Lesmana, C.R.A., Kurniawan, J., Kalista, K.F., Nababan, S.H., and Gani, R.A. (2020). Risk factors of mortality in the patients with hepatocellular carcinoma: A multicenter study in Indonesia. Curr. Probl. Cancer *44*, 100480.
14. Wang, Z., Yu, W., Qiang, Y., Xu, L., Ma, F., Ding, P., Shi, L., Chang, W., Mei, Y., and Ma, X. (2020). LukS-PV Inhibits Hepatocellular Carcinoma Progression by Downregulating HDAC2 Expression. Mol. Ther. Oncolytics *17*, 547–561.
15. Wei, L., Wang, X., Lv, L., Liu, J., Xing, H., Song, Y., Xie, M., Lei, T., Zhang, N., and Yang, M. (2019). The emerging role of microRNAs and long noncoding RNAs in drug resistance of hepatocellular carcinoma. Mol. Cancer *18*, 147.
16. Li, Y., Li, G., Tao, T., Kang, X., Liu, C., Zhang, X., Wang, C., Li, C., and Guo, X. (2019). The μ-opioid receptor (MOR) promotes tumor initiation in hepatocellular carcinoma. Cancer Lett. *453*, 1–9.
17. Kim, Y., Jo, M., Schmidt, J., Luo, X., Prakash, T.P., Zhou, T., Klein, S., Xiao, X., Post, N., Yin, Z., and MacLeod, A.R. (2019). Enhanced Potency of GalNAc-Conjugated Antisense Oligonucleotides in Hepatocellular Cancer Models. Mol. Ther. *27*, 1547–1557.

18. Gong, Z., Yu, J., Yang, S., Lai, P.B.S., and Chen, G.G. (2020). FOX transcription factor family in hepatocellular carcinoma. Biochim. Biophys. Acta Rev. Cancer *1874*, 188376.

19. Yuan, F., Zhang, Y., Ma, L., Cheng, Q., Li, G., and Tong, T. (2017). Enhanced NOLC1 promotes cell senescence and represses hepatocellular carcinoma cell proliferation by disturbing the organization of nucleolus. Aging Cell *16*, 726–737.

20. Zhang, Q., Song, G., Yao, L., Liu, Y., Liu, M., Li, S., and Tang, H. (2018). miR-3928v is induced by HBx via NF-κB/EGR1 and contributes to hepatocellular carcinoma malignancy by down-regulating VDAC3. J. Exp. Clin. Cancer Res. *37*, 14.

21. Ping, Y., Zhou, Y., Hu, J., Pang, L., Xu, C., and Xiao, Y. (2020). Dissecting the Functional Mechanisms of Somatic Copy-Number Alterations Based on Dysregulated ceRNA Networks across Cancers. Mol. Ther. Nucleic Acids *21*, 464–479.

22. Mu, M., Niu, W., Zhang, X., Hu, S., and Niu, C. (2020). LncRNA BCYRN1 inhibits glioma tumorigenesis by competitively binding with miR-619-5p to regulate CUEDC2 expression and the PTEN/AKT/p21 pathway. Oncogene *39*, 6879–6892.

23. Wu, W., Jing, D., Meng, Z., Hu, B., Zhong, B., Deng, X., Jin, X., and Shao, Z. (2020). FGD1 promotes tumor progression and regulates tumor immune response in osteosarcoma via inhibiting PTEN activity. Theranostics *10*, 2859–2871.

24. Tseng, L.-L., Cheng, H.-H., Yeh, T.-S., Huang, S.-C., Syu, Y.-Y., Chuu, C.-P., Yuh, C.-H., Kung, H.-J., and Wang, W.-C. (2020). Targeting the histone demethylase PHF8-mediated PKCα-Src-PTEN axis in HER2-negative gastric cancer. Proc. Natl. Acad. Sci. USA *117*, 24859–24866.

25. Cai, L., Ye, Y., Jiang, Q., Chen, Y., Lyu, X., Li, J., Wang, S., Liu, T., Cai, H., Yao, K., et al. (2020). Author Correction: Epstein-Barr virus-encoded microRNA BART1 induces tumour metastasis by regulating PTEN-dependent pathways in nasopharyngeal carcinoma. Nat. Commun. *11*, 3437.

26. Yang, X.Z., Cheng, T.T., He, Q.J., Lei, Z.Y., Chi, J., Tang, Z., Liao, Q.X., Zhang, H., Zeng, L.S., and Cui, S.Z. (2018). LINC01133 as ceRNA inhibits gastric cancer progression by sponging miR-106a-3p to regulate APC expression and the Wnt/β-catenin pathway. Mol. Cancer *17*, 126.

27. Han, Q., Li, J., Xiong, J., and Song, Z. (2020). Long noncoding RNA LINC00514 accelerates pancreatic cancer progression by acting as a ceRNA of miR-28-5p to upregulate Rap1b expression. J. Exp. Clin. Cancer Res. *39*, 151.

28. Man, X., Piao, C., Lin, X., Kong, C., Cui, X., and Jiang, Y. (2019). USP13 functions as a tumor suppressor by blocking the NF-kB-mediated PTEN downregulation in human bladder cancer. J. Exp. Clin. Cancer Res. *38*, 259.

29. Mi, X., Xu, R., Hong, S., Xu, T., Zhang, W., and Liu, M. (2020). M2 Macrophage-Derived Exosomal lncRNA AFAP1-AS1 and MicroRNA-26a Affect Cell Migration and Metastasis in Esophageal Cancer. Mol. Ther. Nucleic Acids *22*, 779–790.

30. Chen, Z., and Hu, H. (2019). Identification of prognosis biomarkers of prostatic cancer in a cohort of 498 patients from TCGA. Curr. Probl. Cancer *43*, 100503.

31. Tang, G.D., Luo, L.Y., Zhang, J.L., Zhai, D.F., Huang, D.Q., Yin, J., Zhou, Q., Zhang, Q., and Zheng, G.P. (2021). LncRNA LINC01057 promotes mesenchymal differentiation by activating NF-κB signaling in glioblastoma. Cancer Lett. *498*, 152–164.

32. Tong, Y., Yang, L., Yu, C., Zhu, W., Zhou, X., Xiong, Y., Wang, W., Ji, F., He, D., and Cao, X. (2020). Tumor-Secreted Exosomal lncRNA POU3F3 Promotes Cisplatin Resistance in ESCC by Inducing Fibroblast Differentiation into CAFs. Mol. Ther. Oncolytics *18*, 1–13.

33. Zhang, Y., Huang, Y.X., Wang, D.L., Yang, B., Yan, H.Y., Lin, L.H., Li, Y., Chen, J., Xie, L.M., Huang, Y.S., et al. (2020). LncRNA DSCAM-AS1 interacts with YBX1 to promote cancer progression by forming a positive feedback loop that activates FOXA1 transcription network. Theranostics *10*, 10823–10837.

34. Zhou, R., Sun, H., Zheng, S., Zhang, J., Zeng, D., Wu, J., Huang, Z., Rong, X., Bin, J., Liao, Y., et al. (2020). A stroma-related lncRNA panel for predicting recurrence and adjuvant chemotherapy benefit in patients with early-stage colon cancer. J. Cell. Mol. Med. *24*, 3229–3241.

35. Lin, L., Que, Y., Lu, P., Li, H., Xiao, M., Zhu, X., and Li, D. (2020). Chidamide Inhibits Acute Myeloid Leukemia Cell Proliferation by lncRNA VPS9D1-AS1 Downregulation via MEK/ERK Signaling Pathway. Front. Pharmacol. *11*, 569651.

36. Peng, W., Zhang, C., Peng, J., Huang, Y., Peng, C., Tan, Y., Ji, D., Zhang, Y., Zhang, D., Tang, J., et al. (2020). Lnc-FAM84B-4 acts as an oncogenic lncRNA by interacting with protein hnRNPK to restrain MAPK phosphatases-DUSP1 expression. Cancer Lett. *494*, 94–106.

37. Li, Y., Yin, Z., Fan, J., Zhang, S., and Yang, W. (2019). The roles of exosomal miRNAs and lncRNAs in lung diseases. Signal Transduct. Target. Ther. *4*, 47.

38. Mu, Y., Tang, Q., Feng, H., Zhu, L., and Wang, Y. (2020). lncRNA KTN1-AS1 promotes glioma cell proliferation and invasion by negatively regulating miR-505-3p. Oncol. Rep. *44*, 2645–2655.

39. Aleksakhina, S.N., Kashyap, A., and Imyanitov, E.N. (2019). Mechanisms of acquired tumor drug resistance. Biochim. Biophys. Acta Rev. Cancer *1872*, 188310.

40. Zhang, P.F., Pei, X., Li, K.S., Jin, L.N., Wang, F., Wu, J., and Zhang, X.M. (2020). Correction to: Circular RNA circFGFR1 promotes progression and anti-PD-1 resistance by sponging miR-381-3p in non-small cell lung cancer cells. Mol. Cancer *19*, 21.

41. Zhang, X., Niu, W., Mu, M., Hu, S., and Niu, C. (2020). Long non-coding RNA LPP-AS2 promotes glioma tumorigenesis via miR-7-5p/EGFR/PI3K/AKT/c-MYC feedback loop. J. Exp. Clin. Cancer Res. *39*, 196.

42. Shaker, O., Mahfouz, H., Salama, A., and Medhat, E. (2020). Long Non-Coding HULC and miRNA-372 as Diagnostic Biomarkers in Hepatocellular Carcinoma. Rep. Biochem. Mol. Biol. *9*, 230–240.

43. Liu, Z., Wang, Y., Wang, L., Yao, B., Sun, L., Liu, R., Chen, T., Niu, Y., Tu, K., and Liu, Q. (2019). Long non-coding RNA AGAP2-AS1, functioning as a competitive endogenous RNA, upregulates ANXA11 expression by sponging miR-16-5p and promotes proliferation and metastasis in hepatocellular carcinoma. J. Exp. Clin. Cancer Res. *38*, 194.

44. Le Grand, M., Mukha, A., Püschel, J., Valli, E., Kamili, A., Vittorio, O., Dubrovska, A., and Kavallaris, M. (2020). Interplay between MycN and c-Myc regulates radioresistance and cancer stem cell phenotype in neuroblastoma upon glutamine deprivation. Theranostics *10*, 6411–6429.

45. Cancer Genome Atlas Research Network. Electronic address; wheeler@bcm.edu; Cancer Genome Atlas Research Network (2017). Comprehensive and Integrative Genomic Characterization of Hepatocellular Carcinoma. Cell *169*, 1327–1341.e23.

46. Pang, B., Zhu, Y., Ni, J., Thompson, J., Malouf, D., Bucci, J., Graham, P., and Li, Y. (2020). Extracellular vesicles: the next generation of biomarkers for liquid biopsy-based prostate cancer diagnosis. Theranostics *10*, 2309–2326.

47. Min, L., Zhu, S., Wei, R., Zhao, Y., Liu, S., Li, P., and Zhang, S. (2020). Integrating SWATH-MS Proteomics and Transcriptome Analysis Identifies CHI3L1 as a Plasma Biomarker for Early Gastric Cancer. Mol. Ther. Oncolytics *17*, 257–266.

48. Heller, L., Thinard, R., Chevalier, M., Arpag, S., Jing, Y., Greferath, R., Heller, R., and Nicolau, C. (2020). Secretion of proteins and antibody fragments from transiently transfected endothelial progenitor cells. J. Cell. Mol. Med. *24*, 8772–8778.

49. Wang, J., Zhu, S., Meng, N., He, Y., Lu, R., and Yan, G.R. (2019). ncRNA-Encoded Peptides or Proteins and Cancer. Mol. Ther. *27*, 1718–1725.

50. Ma, Y.S., Chu, K.J., Ling, C.C., Wu, T.M., Zhu, X.C., Liu, J.B., Yu, F., Li, Z.Z., Wang, J.H., Gao, Q.X., et al. (2020). Long Noncoding RNA OIP5-AS1 Promotes the Progression of Liver Hepatocellular Carcinoma via Regulating the hsa-miR-26a-3p/EPHA2 Axis. Mol. Ther. Nucleic Acids *21*, 229–241.

51. Chen, Z., Zheng, Z., Feng, L., Huo, Z., Huang, L., Fu, M., Chen, Q., Ke, Y., Yang, J., and Hou, B. (2020). Overexpression of miR-382 Sensitizes Hepatocellular Carcinoma Cells to γδ T Cells by Inhibiting the Expression of c-FLIP. Mol. Ther. Oncolytics *18*, 467–475.

52. Teng, F., Zhang, J.X., Chang, Q.M., Wu, X.B., Tang, W.G., Wang, J.F., Feng, J.F., Zhang, Z.P., and Hu, Z.Q. (2020). LncRNA MYLK-AS1 facilitates tumor progression and angiogenesis by targeting miR-424-5p/E2F7 axis and activating VEGFR-2 signaling pathway in hepatocellular carcinoma. J. Exp. Clin. Cancer Res. *39*, 235.

53. Fergusson, D.A., Wesch, N.L., Leung, G.J., MacNeil, J.L., Conic, I., Presseau, J., Cobey, K.D., Diallo, J.S., Auer, R., Kimmelman, J., et al. (2019). Assessing the Completeness of Reporting in Preclinical Oncolytic Virus Therapy Studies. Mol. Ther. Oncolytics *14*, 179–187.

54. Ma, Y.S., Wang, X.F., Yu, F., Wu, T.M., Liu, J.B., Zhang, Y.J., Xia, Q., Jiang, Z.Y., Lin, Q.L., and Fu, D. (2020). Inhibition of USP14 and UCH37 deubiquitinating activity

by b-AP15 as a potential therapy for tumors with p53 deficiency. Signal Transduct. Target. Ther. *5*, 30.

55. Xu, H., Chen, G.F., Ma, Y.S., Zhang, H.W., Zhou, Y., Liu, G.H., Chen, D.Y., Ping, J., Liu, Y.H., Mou, X., and Fu, D. (2020). Hepatic Proteomic Changes and Sirt1/AMPK Signaling Activation by Oxymatrine Treatment in Rats With Non-alcoholic Steatosis. Front. Pharmacol. *11*, 216.

56. Zhao, B., Ke, K., Wang, Y., Wang, F., Shi, Y., Zheng, X., Yang, X., Liu, X., and Liu, J. (2020). HIF-1α and HDAC1 mediated regulation of FAM99A-miR92a signaling contributes to hypoxia induced HCC metastasis. Signal Transduct. Target. Ther. *5*, 118.

57. Xiao, Y., Najeeb, R.M., Ma, D., Yang, K., Zhong, Q., and Liu, Q. (2019). Upregulation of CENPM promotes hepatocarcinogenesis through mutiple mechanisms. J. Exp. Clin. Cancer Res. *38*, 458.

58. Tam, B.Y., Chiu, K., Chung, H., Bossard, C., Nguyen, J.D., Creger, E., Eastman, B.W., Mak, C.C., Ibanez, M., Ghias, A., et al. (2020). The CLK inhibitor SM08502 induces anti-tumor activity and reduces Wnt pathway gene expression in gastrointestinal cancer models. Cancer Lett. *473*, 186–197.

59. Li, M., Shao, J., Guo, Z., Jin, C., Wang, L., Wang, F., Jia, Y., Zhu, Z., Zhang, Z., Zhang, F., et al. (2020). Novel mitochondrion-targeting copper(II) complex induces HK2 malfunction and inhibits glycolysis via Drp1-mediating mitophagy in HCC. J. Cell. Mol. Med. *24*, 3091–3107.

60. Chen, Y., Zhao, H., Li, H., Feng, X., Tang, H., Qiu, C., Zhang, J., and Fu, B. (2020). LINC01234/MicroRNA-31-5p/MAGEA3 Axis Mediates the Proliferation and Chemoresistance of Hepatocellular Carcinoma Cells. Mol. Ther. Nucleic Acids *19*, 168–178.

61. Shao, Y., Song, X., Jiang, W., Chen, Y., Ning, Z., Gu, W., and Jiang, J. (2019). MicroRNA-621 acts as a tumor radiosensitizer by directly targeting SETDB1 in hepatocellular carcinoma. Mol. Ther. *27*, 355–364.

62. Zeng, C., Liu, S., Lu, S., Yu, X., Lai, J., Wu, Y., Chen, S., Wang, L., Yu, Z., Luo, G., and Li, Y. (2018). The c-Myc-regulated lncRNA NEAT1 and paraspeckles modulate imatinib-induced apoptosis in CML cells. Mol. Cancer *17*, 130.

63. van den Ende, T., van den Boorn, H.G., Hoonhout, N.M., van Etten-Jamaludin, F.S., Meijer, S.L., Derks, S., de Gruijl, T.D., Bijlsma, M.F., van Oijen, M.G.H., and van Laarhoven, H.W.M. (2020). Priming the tumor immune microenvironment with chemo(radio)therapy: A systematic review across tumor types. Biochim. Biophys. Acta Rev. Cancer *1874*, 188386.

64. Wang, H.L., Liu, P.F., Yue, J., Jiang, W.H., Cui, Y.L., Ren, H., Wang, H., Zhuang, Y., Liu, Y., Jiang, D., et al. (2020). Somatic gene mutation signatures predict cancer type and prognosis in multiple cancers with pan-cancer 1000 gene panel. Cancer Lett. *470*, 181–190.

65. Cheng, J., Zhuo, H., Wang, L., Zheng, W., Chen, X., Hou, J., Zhao, J., and Cai, J. (2020). Identification of the Combinatorial Effect of miRNA Family Regulatory Network in Different Growth Patterns of GC. Mol. Ther. Oncolytics *17*, 531–546.

66. Tian, X., Yu, H., Li, D., Jin, G.J., Dai, S.D., Gong, P.C., Kong, C.C., and Wang, X.J. (2021). The miR-5694/AF9/Snail axis provides metastatic advantages and a therapeutic target in basal-like breast cancer. Mol. Ther. *29*, 1239–1257.

67. Hewes, A.M., Sansbury, B.M., Barth, S., Tarcic, G., and Kmiec, E.B. (2020). gRNA Sequence Heterology Tolerance Catalyzed by CRISPR/Cas in an In Vitro Homology-Directed Repair Reaction. Mol. Ther. Nucleic Acids *20*, 568–579.

68. Bao, W.D., Zhou, X.T., Zhou, L.T., Wang, F., Yin, X., Lu, Y., Zhu, L.Q., and Liu, D. (2020). Targeting miR-124/Ferroportin signaling ameliorated neuronal cell death through inhibiting apoptosis and ferroptosis in aged intracerebral hemorrhage murine model. Aging Cell *19*, e13235.

69. Wang, S.M., Pang, J., Zhang, K.J., Zhou, Z.Y., and Chen, F.Y. (2021). lncRNA MIR503HG inhibits cell proliferation and promotes apoptosis in TNBC cells via the miR-224-5p/HOXA9 axis. Mol. Ther. Oncolytics *21*, 62–73.

70. Song, X., Xu, P., Meng, C., Song, C., Blackwell, T.S., Li, R., Li, H., Zhang, J., and Lv, C. (2019). lncITPF Promotes Pulmonary Fibrosis by Targeting hnRNP-L Depending on Its Host Gene ITGBL1. Mol. Ther. *27*, 380–393.

71. Qi, W.Y., Mao, X.B., He, Y.B., and Xiao, C.H. (2020). Long non-coding RNA LINC00858 promotes cells proliferation and invasion through the miR-153-3p/Rabl3 axis in hepatocellular carcinoma. Eur. Rev. Med. Pharmacol. Sci. *24*, 9343–9352.

72. Cui, J., Wang, L., Zhong, W., Chen, Z., Chen, J., Yang, H., and Liu, G. (2020). Identification and validation of methylation-driven genes prognostic signature for recurrence of laryngeal squamous cell carcinoma by integrated bioinformatics analysis. Cancer Cell Int. *20*, 472.

73. Jiang, Y., and Luo, Y. (2020). LINC01354 Promotes Osteosarcoma Cell Invasion by Up-regulating Integrin β1. Arch. Med. Res. *51*, 115–123.

74. Li, R., Yang, Y.E., Yin, Y.H., Zhang, M.Y., Li, H., and Qu, Y.Q. (2019). Methylation and transcriptome analysis reveal lung adenocarcinoma-specific diagnostic biomarkers. J. Transl. Med. *17*, 324.

75. Li, J., He, M., Xu, W., and Huang, S. (2019). LINC01354 interacting with hnRNP-D contributes to the proliferation and metastasis in colorectal cancer through activating Wnt/β-catenin signaling pathway. J. Exp. Clin. Cancer Res. *38*, 161.

76. Yang, G., Yang, C., She, Y., Shen, Z., and Gao, P. (2019). LINC01354 enhances the proliferation and invasion of lung cancer cells by regulating miR-340-5p/ATF1 signaling pathway. Artif. Cells Nanomed. Biotechnol. *47*, 3737–3744.

77. Ma, X., Mo, M., Tan, H.J.J., Tan, C., Zeng, X., Zhang, G., Huang, D., Liang, J., Liu, S., and Qiu, X. (2020). LINC02499, a novel liver-specific long non-coding RNA with potential diagnostic and prognostic value, inhibits hepatocellular carcinoma cell proliferation, migration, and invasion. Hepatol. Res. *50*, 726–740.

78. Hu, J., Chen, Z., Bao, L., Zhou, L., Hou, Y., Liu, L., Xiong, M., Zhang, Y., Wang, B., Tao, Z., and Chan, K. (2020). Single-Cell Transcriptome Analysis Reveals Intratumoral Heterogeneity in ccRCC, which Results in Different Clinical Outcomes. Mol. Ther. *28*, 1658–1672.

79. Kawamura, E., Maruyama, M., Abe, J., Sudo, A., Takeda, A., Takada, S., Yokota, T., Kinugawa, S., Harashima, H., and Yamada, Y. (2020). Validation of Gene Therapy for Mutant Mitochondria by Delivering Mitochondrial RNA Using a MITO-Porter. Mol. Ther. Nucleic Acids *20*, 687–698.

80. Elsedawy, N.B., Nace, R.A., Russell, S.J., and Schulze, A.J. (2020). Oncolytic Activity of Targeted Picornaviruses Formulated as Synthetic Infectious RNA. Mol. Ther. Oncolytics *17*, 484–495.

81. Gupta, S.C., Awasthee, N., Rai, V., Chava, S., Gunda, V., and Challagundla, K.B. (2020). Long non-coding RNAs and nuclear factor-κB crosstalk in cancer and other human diseases. Biochim. Biophys. Acta Rev. Cancer *1873*, 188316.

82. Iwai, N., Yasui, K., Tomie, A., Gen, Y., Terasaki, K., Kitaichi, T., Soda, T., Yamada, N., Dohi, O., Seko, Y., et al. (2018). Oncogenic miR-96-5p inhibits apoptosis by targeting the caspase-9 gene in hepatocellular carcinoma. Int. J. Oncol. *53*, 237–245.

83. Chen, Z., Xiang, B., Qi, L., Zhu, S., and Li, L. (2020). miR-221-3p promotes hepatocellular carcinogenesis by downregulating O6-methylguanine-DNA methyltransferase. Cancer Biol. Ther. *21*, 915–926.

84. Yang, J., Cui, R., and Liu, Y. (2020). MicroRNA-212-3p inhibits paclitaxel resistance through regulating epithelial-mesenchymal transition, migration and invasion by targeting ZEB2 in human hepatocellular carcinoma. Oncol. Lett. *20*, 23.

85. Ghosh, S., Bhowmik, S., Majumdar, S., Goswami, A., Chakraborty, J., Gupta, S., Aggarwal, S., Ray, S., Chatterjee, R., Bhattacharyya, S., et al. (2020). The exosome encapsulated microRNAs as circulating diagnostic marker for hepatocellular carcinoma with low alpha-fetoprotein. Int. J. Cancer *147*, 2934–2947.

86. Liu, Y., Cao, Y., Cai, W., Wu, L., Zhao, P., and Liu, X.G. (2020). Aberrant expression of two miRNAs promotes proliferation, hepatitis B virus amplification, migration and invasion of hepatocellular carcinoma cells: evidence from bioinformatic analysis and experimental validation. PeerJ *8*, e9100.

87. Wang, X., Liao, X., Huang, K., Zeng, X., Liu, Z., Zhou, X., Yu, T., Yang, C., Yu, L., Wang, Q., et al. (2019). Clustered microRNAs hsa-miR-221-3p/hsa-miR-222-3p and their targeted genes might be prognostic predictors for hepatocellular carcinoma. J. Cancer *10*, 2520–2533.

88. Shen, S.M., Ji, Y., Zhang, C., Dong, S.S., Yang, S., Xiong, Z., Ge, M.K., Yu, Y., Xia, L., Guo, M., et al. (2018). Nuclear PTEN safeguards pre-mRNA splicing to link Golgi apparatus for its tumor suppressive role. Nat. Commun. *9*, 2392.

89. Chen, J., Liu, A., Lin, Z., Wang, B., Chai, X., Chen, S., Lu, W., Zheng, M., Cao, T., Zhong, M., et al. (2020). Downregulation of the circadian rhythm regulator HLF promotes multiple-organ distant metastases in non-small cell lung cancer through PPAR/NF-κb signaling. Cancer Lett. *482*, 56–71.

90. Huang, Z., Yu, H., Du, G., Han, L., Huang, X., Wu, D., Han, X., Xia, Y., Wang, X., and Lu, C. (2021). Enhancer RNA lnc-CES1-1 inhibits decidual cell migration by interacting with RNA-binding protein FUS and activating PPARγ in URPL. Mol. Ther. Nucleic Acids *24*, 104–112.

91. Zeng, J., Tang, Z., Zhang, Y., Tong, X., Dou, J., Gao, L., Ding, S., and Lu, J. (2021). Ozonated autohemotherapy elevates PPAR-γ expression in $CD4^+$ T cells and serum HDL-C levels, a potential immunomodulatory mechanism for treatment of psoriasis. Am. J. Transl. Res. *13*, 349–359.

92. Yu, Q., Cheng, P., Wu, J., and Guo, C. (2021). PPARγ/NF-κB and TGF-β1/Smad pathway are involved in the anti-fibrotic effects of levo-tetrahydropalmatine on liver fibrosis. J. Cell. Mol. Med. *25*, 1645–1660.

93. Chen, H.X., Li, M.Y., Jiang, Y.Y., Hou, H.T., Wang, J., Liu, X.C., Yang, Q., and He, G.W. (2020). Role of the PPAR pathway in atrial fibrillation associated with heart valve disease: transcriptomics and proteomics in human atrial tissue. Signal Transduct. Target. Ther. *5*, 4.

94. Miao, Y., Zheng, Y., Geng, Y., Yang, L., Cao, N., Dai, Y., and Wei, Z. (2021). The role of GLS1-mediated glutaminolysis/2-HG/H3K4me3 and GSH/ROS signals in Th17 responses counteracted by PPARγ agonists. Theranostics *11*, 4531–4548.

95. Li, Z., Lou, Y., Tian, G., Wu, J., Lu, A., Chen, J., Xu, B., Shi, J., and Yang, J. (2019). Discovering master regulators in hepatocellular carcinoma: one novel MR, SEC14L2 inhibits cancer cells. Aging (Albany NY) *11*, 12375–12411.

96. Chen, C., Cai, Z., Zhuo, Y., Xi, M., Lin, Z., Jiang, F., Liu, Z., Wan, Y., Zheng, Y., Li, J., et al. (2020). Overexpression of SLC6A1 associates with drug resistance and poor prognosis in prostate cancer. BMC Cancer *20*, 289.

97. Kudo, M., Han, K.H., Ye, S.L., Zhou, J., Huang, Y.H., Lin, S.M., Wang, C.K., Ikeda, M., Chan, S.L., Choo, S.P., et al. (2020). A Changing Paradigm for the Treatment of Intermediate-Stage Hepatocellular Carcinoma: Asia-Pacific Primary Liver Cancer Expert Consensus Statements. Liver Cancer *9*, 245–260.

98. Gagan, J., and Van Allen, E.M. (2015). Next-generation sequencing to guide cancer therapy. Genome Med. *7*, 80.

99. Li, S., Cui, Z., Gu, J., Wang, Y., Tang, S., and Chen, J. (2021). Effect of porcine corneal stromal extract on keratocytes from SMILE-derived lenticules. J. Cell. Mol. Med. *25*, 1207–1220.

100. Shannon, P., Markiel, A., Ozier, O., Baliga, N.S., Wang, J.T., Ramage, D., Amin, N., Schwikowski, B., and Ideker, T. (2003). Cytoscape: a software environment for integrated models of biomolecular interaction networks. Genome Res. *13*, 2498–2504.

101. Cerami, E., Gao, J., Dogrusoz, U., Gross, B.E., Sumer, S.O., Aksoy, B.A., Jacobsen, A., Byrne, C.J., Heuer, M.L., Larsson, E., et al. (2012). The cBio cancer genomics portal: an open platform for exploring multidimensional cancer genomics data. Cancer Discov. *2*, 401–404.

102. Gao, J., Aksoy, B.A., Dogrusoz, U., Dresdner, G., Gross, B., Sumer, S.O., Sun, Y., Jacobsen, A., Sinha, R., Larsson, E., et al. (2013). Integrative analysis of complex cancer genomics and clinical profiles using the cBioPortal. Sci. Signal. *6*, pl1.

103. Han, H.J., Sung, J.Y., Kim, S.H., Yun, U.J., Kim, H., Jang, E.J., Yoo, H.E., Hong, E.K., Goh, S.H., Moon, A., et al. (2021). Fibronectin regulates anoikis resistance via cell aggregate formation. Cancer Lett. *508*, 59–72.

104. Cai, Y., Wu, G., Peng, B., Li, J., Zeng, S., Yan, Y., and Xu, Z. (2021). Expression and molecular profiles of the AlkB family in ovarian serous carcinoma. Aging (Albany NY) *13*, 9679–9692.

105. Li, B., Severson, E., Pignon, J.C., Zhao, H., Li, T., Novak, J., Jiang, P., Shen, H., Aster, J.C., Rodig, S., et al. (2016). Comprehensive analyses of tumor immunity: implications for cancer immunotherapy. Genome Biol. *17*, 174.

106. Chandrashekar, D.S., Bashel, B., Balasubramanya, S.A.H., Creighton, C.J., Ponce-Rodriguez, I., Chakravarthi, B.V.S.K., and Varambally, S. (2017). UALCAN: A Portal for Facilitating Tumor Subgroup Gene Expression and Survival Analyses. Neoplasia *19*, 649–658.

107. Zheng, Y., Tang, L., Chen, G., and Liu, Z. (2020). Comprehensive Bioinformatics Analysis of Key Methyltransferases and Demethylases for Histone Lysines in Hepatocellular Carcinoma. Technol. Cancer Res. Treat. *19*, 1533033820983284.

108. Guo, J., Zhu, J., Wang, Q., Wang, J., and Jia, Y. (2021). Comparative Efficacy of Seven Kinds of Chinese Medicine Injections in Acute Lung Injury and Acute Respiratory Distress Syndrome: A Network Meta-analysis of Randomized Controlled Trials. Front. Pharmacol. *12*, 627751.

109. Yu, C., Hao, X., Zhang, S., Hu, W., Li, J., Sun, J., and Zheng, M. (2019). Characterization of the prognostic values of the *NDRG* family in gastric cancer. Therap. Adv. Gastroenterol. *12*, 1756284819858507.

110. Koch, A., De Meyer, T., Jeschke, J., and Van Criekinge, W. (2015). MEXPRESS: visualizing expression, DNA methylation and clinical TCGA data. BMC Genomics *16*, 636.

111. Koch, A., Jeschke, J., Van Criekinge, W., van Engeland, M., and De Meyer, T. (2019). MEXPRESS update 2019. Nucleic Acids Res. *47* (W1), W561–W565.

112. Li, J.Y., Li, C.J., Lin, L.T., and Tsui, K.H. (2020). Multi-Omics Analysis Identifying Key Biomarkers in Ovarian Cancer. Cancer Contr. *27*, 1073274820976671.

113. Mulder, J., Wernérus, H., Shi, T.J., Pontén, F., Hober, S., Uhlén, M., and Hökfelt, T. (2007). Systematically generated antibodies against human gene products: high throughput screening on sections from the rat nervous system. Neuroscience *146*, 1689–1703.

114. Dammeyer, P., and Arnér, E.S. (2011). Human Protein Atlas of redox systems - what can be learnt? Biochim. Biophys. Acta *1810*, 111–138.

115. Uhlen, M., Zhang, C., Lee, S., Sjöstedt, E., Fagerberg, L., Bidkhori, G., Benfeitas, R., Arif, M., Liu, Z., Edfors, F., et al. (2017). A pathology atlas of the human cancer transcriptome. Science *357*, eaan2507.

OMTN, Volume *24*

Supplemental information

Construction of a Myc-associated ceRNA network reveals a prognostic signature in hepatocellular carcinoma

Dan-Dan Zhang, Yi Shi, Ji-Bin Liu, Xiao-Li Yang, Rui Xin, Hui-Min Wang, Pei-Yao Wang, Cheng-You Jia, Wen-Jie Zhang, Yu-Shui Ma, and Da Fu

Table S1. Correlation analysis between SEC14L2, SLC6A1 and biomarkers of immune cells in HCC

Description	Gene markers	SEC14L2		SLC6A1	
		Cor	P	Cor	P
CD8+ T cell	CD8A	-0.211	4.32e-05	-0.129	1.32e-02
	CD8B	-0.259	4.28e-07	-0.239	3.19e-06
T cell (general)	CD3D	-0.411	0e+00	-0.3	4.3e-09
	CD3E	-0.326	1.54e-10	-0.195	1.6e-04
	CD2	-0.346	9.7e-12	-0.203	8.81e-05
B cell	CD19	-0.24	2.86e-06	-0.121	1.96e-02
	CD79A	-0.274	8.33e-08	-0.136	8.84e-03
Monocyte	CD86	-0.282	3.78e-08	-0.235	5.04e-06
	CD115 (CSF1R)	-0.194	1.68e-04	-0.253	8.76e-07
TAM	CCL2	-0.075	1.48e-01	-0.133	1.05e-02
	CD68	-0.293	1.07e-08	-0.292	1.21e-08
	IL10	-0.23	7.39e-06	-0.156	2.51e-03
M1 Macrophage	INOS (NOS2)	0.174	7.9e-04	0.243	2.15e-06
	IRF5	-0.111	3.23e-02	0.139	7.13e-03
	COX2 (PTGS2)	-0.185	3.48e-04	-0.099	5.79e-02
M2 Macrophage	CD163	0.008	8.73e-01	-0.061	2.38e-01
	VSIG4	-0.053	3.1e-01	-0.15	3.92e-03

	MS4A4A	-0.109	3.63e-02	-0.148	4.37e-03
Neutrophils	CD66b (CEACAM8)	-0.064	2.19e-01	-0.073	1.61e-01
	CD11b (ITGAM)	-0.147	4.45e-03	-0.14	7.09e-03
	CCR7	-0.206	6.28e-05	-0.042	4.2e-01
Natural killer cell	KIR2DL1	0.049	3.46e-01	-0.004	9.37e-01
	KIR2DL3	-0.002	9.67e-01	0.003	9.59e-01
	KIR2DL4	-0.079	1.31e-01	-0.171	9.55e-04
	KIR3DL1	0.085	1.04e-01	0.039	4.59e-01
	KIR3DL2	-0.064	2.2e-01	-0.05	3.33e-01
	KIR3DL3	-0.036	4.88e-01	-0.003	9.58e-01
	KIR2DS4	0.02	6.95e-01	0.017	7.48e-01
Dendritic cell	HLA-DPB1	-0.195	1.67e-04	-0.256	6.23e-07
	HLA-DQB1	-0.209	5.37e-05	-0.23	8.06e-06
	HLA-DRA	-0.131	1.16e-02	-0.208	5.7e-05
	HLA-DPA1	-0.144	5.48e-03	-0.164	1.6e-03
	BDCA-1(CD1C)	-0.186	3.1e-04	-0.043	4.12e-01
	BDCA-4(NRP1)	-0.093	7.41e-02	-0.046	3.79e-01
	CD11c(ITGAX)	-0.274	9.59e-08	-0.156	2.6e-03
Th1	T-bet (TBX21)	-0.087	9.45e-02	-0.071	1.7e-01
	STAT4	-0.285	2.53e-08	0.006	9.05e-01
	STAT1	-0.185	3.59e-04	0.05	3.41e-01

	IFN-γ (IFNG)	-0.169	1.08e-03	-0.149	4.01e-03
	TNF-α (TNF)	-0.236	4.31e-06	-0.146	4.92e-03
Th2	GATA3	-0.302	3.04e-09	-0.145	5e-03
	STAT6	0.119	2.2e-02	0.159	2.19e-03
	STAT5A	-0.134	9.66e-03	-0.09	8.2e-02
	IL13	0.056	2.84e-01	0.189	2.56e-04
Tfh	BCL6	0.104	4.53e-02	0.081	1.19e-01
	IL21	-0.1	5.47e-02	-0.026	6.24e-01
Th17	STAT3	-0.035	5.01e-01	0.075	1.5e-01
	IL17A	-0.061	2.44e-01	0.04	4.43e-01
Treg	FOXP3	0.198	1.27e-04	0.276	6.83e-08
	CCR8	-0.201	9.49e-05	0.044	3.97e-01
	STAT5B	0.214	3.45e-05	0.423	0e+00
	TGF β (TGFB1)	-0.43	0e+00	-0.243	2.34e-06
T cell exhaustion	PD-1 (PDCD1)	-0.41	1.83e-16	-0.245	1.75e-06
	CTLA4	-0.441	4.73e-19	-0.301	3.4e-09
	LAG3	-0.209	4.97e-05	-0.159	2.08e-03
	TIM-3 (HAVCR2)	-0.345	1.19e-11	-0248	1.49e-06
	GZMB	-0.105	4.34e-02	-0.196	1.48e-04

Table S2. Correlation analysis between SEC14L2 and biomarkers of T cell (general), monocyte, TAM and T cell exhaustion in GEPIA.

Description	Gene markers	LIHC(SEC14L2)			
		Tumor		Normal	
		R	P	R	P
T cell (general)	CD3D	-0.22	***	-0.13	0.37
	CD3E	-0.16	*	-0.092	0.52
	CD2	-0.17	**	-0.11	0.44
Monocyte	CD86	-0.2	***	-0.16	0.26
	CD115	-0.14	*	-0.05	0.73
TAM	CCL2	-0.11	0.035	-0.24	0.098
	CD68	-0.14	*	-0.076	0.6
	IL10	-0.085	0.1	0.17	0.24
T cell exhaustion	PD-1	-0.15	*	0.0016	0.99
	CTLA4	-0.24	***	-0.088	0.54
	LAG3	-0.16	*	0.00031	1
	TIM-3	-0.16	*	-0.24	0.086
	GZMB	-0.15	*	-0.085	0.56

*P<0.01, ** P<0.001, *** P<0.0001.

Table S3. Univariate analysis of overall survival in LIHC patients stratified based on clinical characteristics.

Factor	Variable	N	SLC6A1		SEC14L2		Overall survival		
			median	P value	median	P value	Months (median)	95%CI	P value (Log-rank test)
Age	≧60	200	19.91	0.511	18.99	0.001	21.05	23.21-29.95	0.272
	<60	169	19.24		9.45		18.53	23.28-30.68	
Gender	male	248	18.05	0.137	15.26	0.001	19.07	23.21-29.05	0.257
	female	121	24.1		8.95		21.07	23.39-32.75	
BMI	≦18.5	21	12	0.005	8.32	0.266	19.57	14.04-43.23	0.133
	18.5-23.9	135	17.1		11.38		15.63	19.31-26.56	
	24-27.9	80	21.93		14.07		21.45	25.87-37.91	
	≧28	97	21.63		16.9		23.27	25.22-34.93	
Race	yellow	157	17.64	0.197	12.03	0.539	17.83	22.28-29.56	0.196
	white	184	21.28		13.41		22.22	24.65-32.17	
	other	28	15.7		18.66		16.65	13.53-27.80	

TNM stage	I-II	255	20.21	0.032	15.13	0.062	20.27	25.49-31.63	0
	III-IV	90	16.51		8.87		13.67	17.44-27.31	
	Unknown	24	28.31		20.48		24.52	17.05-31.18	
Diameter	< 5cm	273	20.26	0.022	15.13	0.022	19.87	24.95-30.90	0.004
	≥ 5cm	93	17.27		9.22		18.6	19.13-28.23	
	Negative	251	18.5	0.149	3.44	0.58	20.27	26.33-32.95	0.001
Lymph-node metastasis	Positive	4	27.45		4.03		20.55	5.33-34.67	
	Unknown	113	21.24		15.15		18.5	17.67-23.98	
Distant metastasis	Negative	265	18.5	0.096	12.16	0.094	20.03	25.38-31.61	0.031
	Positive	4	5.13		2.71		6.6	10.81-31.65	
	Unknown	100	21.94		16.26		18.57	18.99-26.66	
Prior malignancy	Yes	35	20.09	0.432	24.98	0.172	20.03	15.73-28.97	0.013
	No	334	19.54		12.59		19.58	24.57-29.88	

Table S4. Univariate analysis of overall survival in LIHC patients stratified based on clinical Characteristics

Factor	Variable	N	LINC02499		Overall survival		
			median	P value	Months (median)	95%CI	P value (Log-rank test)
Age	≧60	200	2.67	0.477	21.05	23.21-29.95	0.272
	<60	169	2.78		18.53	23.28-30.68	
Gender	male	248	2.88	0.683	19.07	23.21-29.05	0.257
	female	121	2.43		21.07	23.39-32.75	
BMI	≦18.5	21	2.41	0.04	19.57	14.04-43.23	0.133
	18.5-23.9	135	2.09		15.63	19.31-26.56	
	24-27.9	80	2.7		21.45	25.87-37.91	
	≧28	97	3.3		23.27	25.22-34.93	
Race	yellow	157	2.16	0.013	17.83	22.28-29.56	0.196
	white	184	3.28		22.22	24.65-32.17	
	other	28	2.6		16.65	13.53-27.80	
TNM stage	Ⅰ-Ⅱ	255	2.53	0.767	20.27	25.49-31.63	0
	Ⅲ-Ⅳ	90	3.31		13.67	17.44-27.31	
	Unknown	24	2.8		24.52	17.05-31.18	

Diameter	< 5cm	273	2.6	0.38	19.87	24.95-30.90	0.004
	≥ 5cm	93	3.32		18.6	19.13-28.23	
Lymph-node metastasis	Negative	251	2.42	0.048	20.27	26.33-32.95	0.001
	Positive	4	3.26		20.55	5.33-34.67	
	Unknown	113	3.52		18.5	17.67-23.98	
Distant metastasis	Negative	265	2.64	0.307	20.03	25.38-31.61	0.031
	Positive	4	4.36		6.6	10.81-31.65	
	Unknown	100	2.88		18.57	18.99-26.66	
Prior malignancy	Yes	35	2.1	0.297	20.03	15.73-28.97	0.013
	No	334	2.79		19.58	24.57-29.88	

Supplemental figure and legends

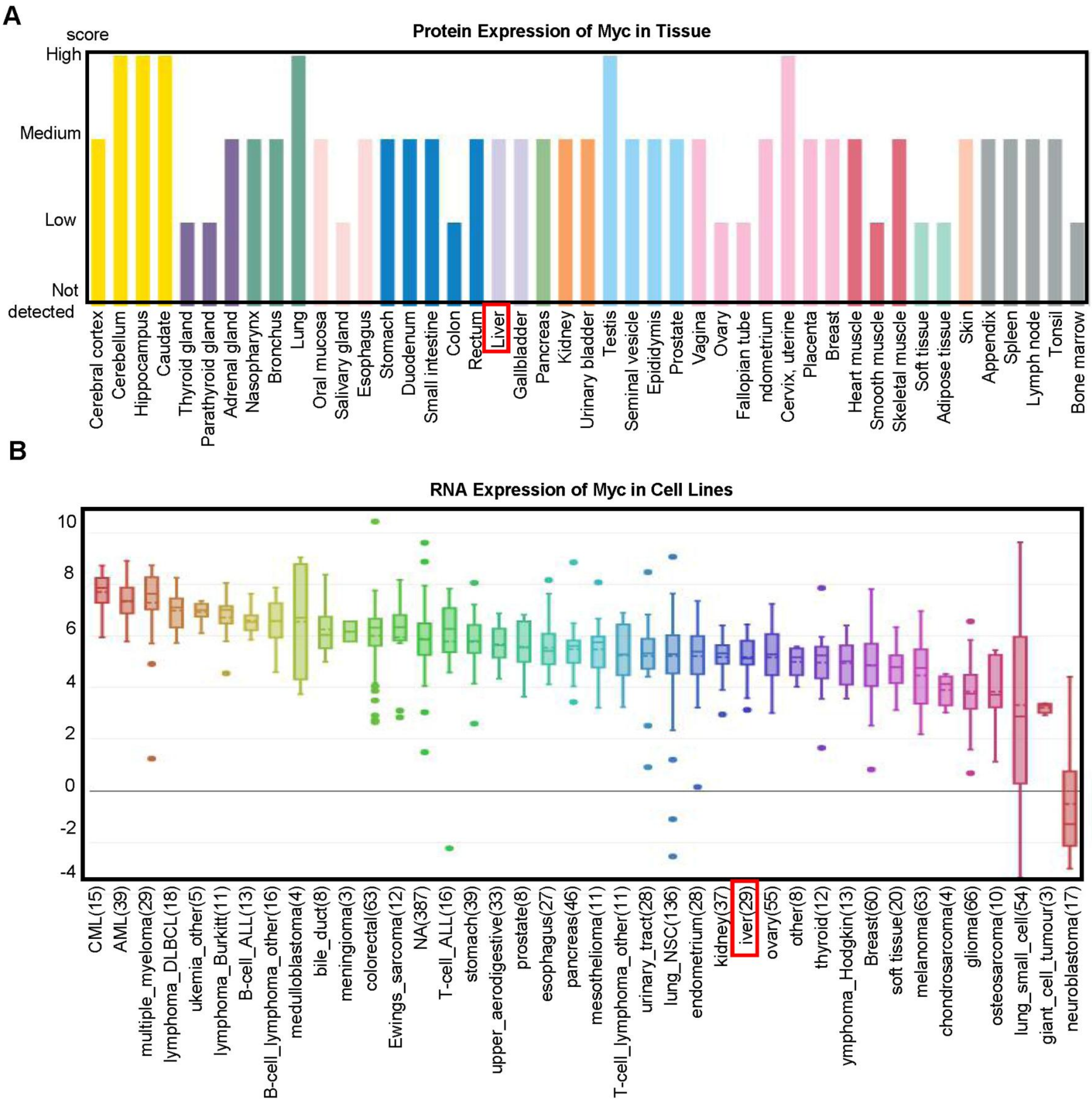

Fig. S1. Myc expression. (A) Expression of MYC in various normal organ tissues according to the HPA database. (B) Expression of Myc in cancer cell lines in CCLE.

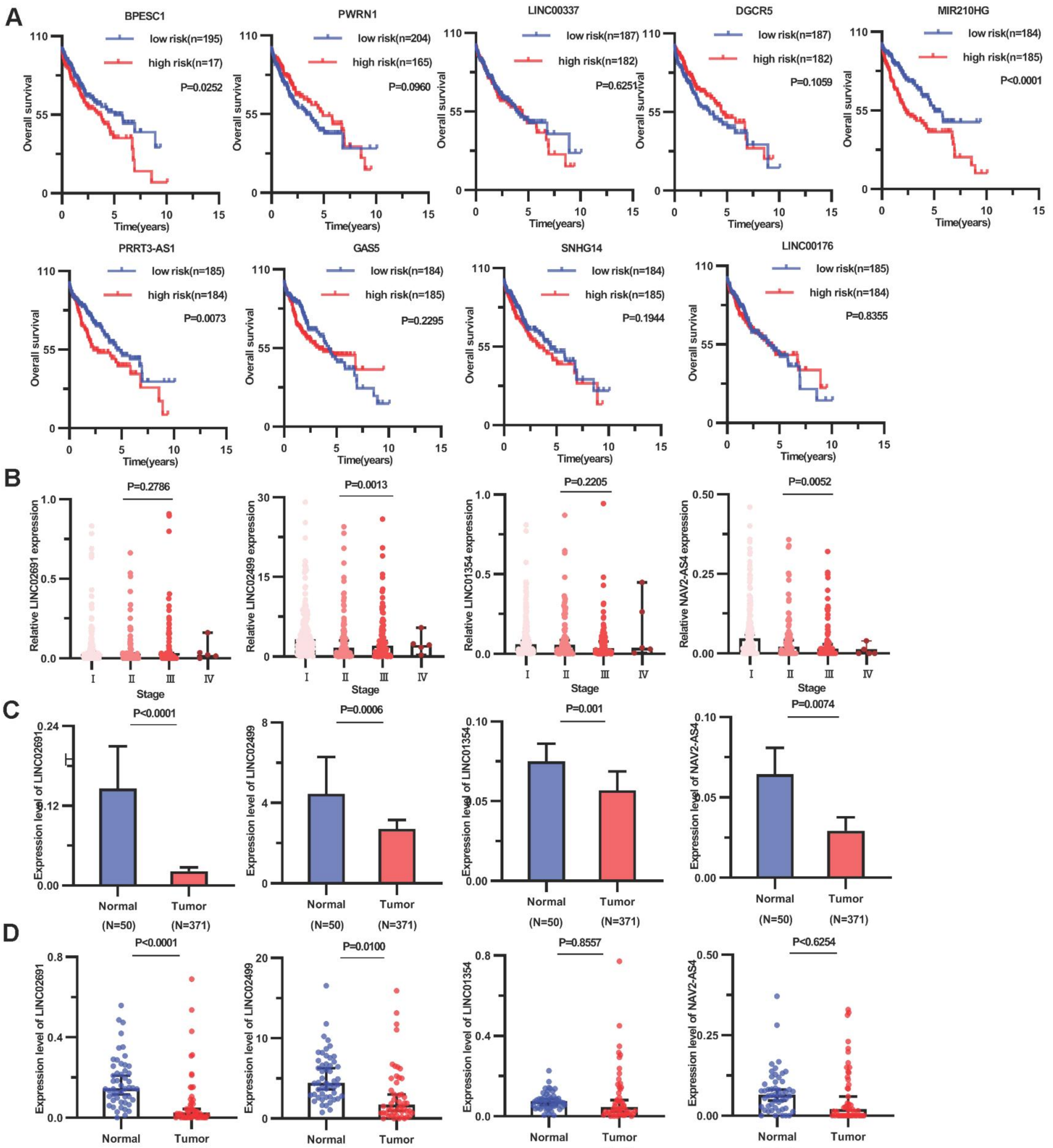

Fig. S2 Analysis of LINC02691, LINC02499, LINC01354, and NAV2-AS4. (A) Survival analysis. (B) TNM stage. (C) Differential expression in 371 HCC tissues and 50 adjacent normal tissues. (D) Validation of 50 paired cancer and paracancer tissues.

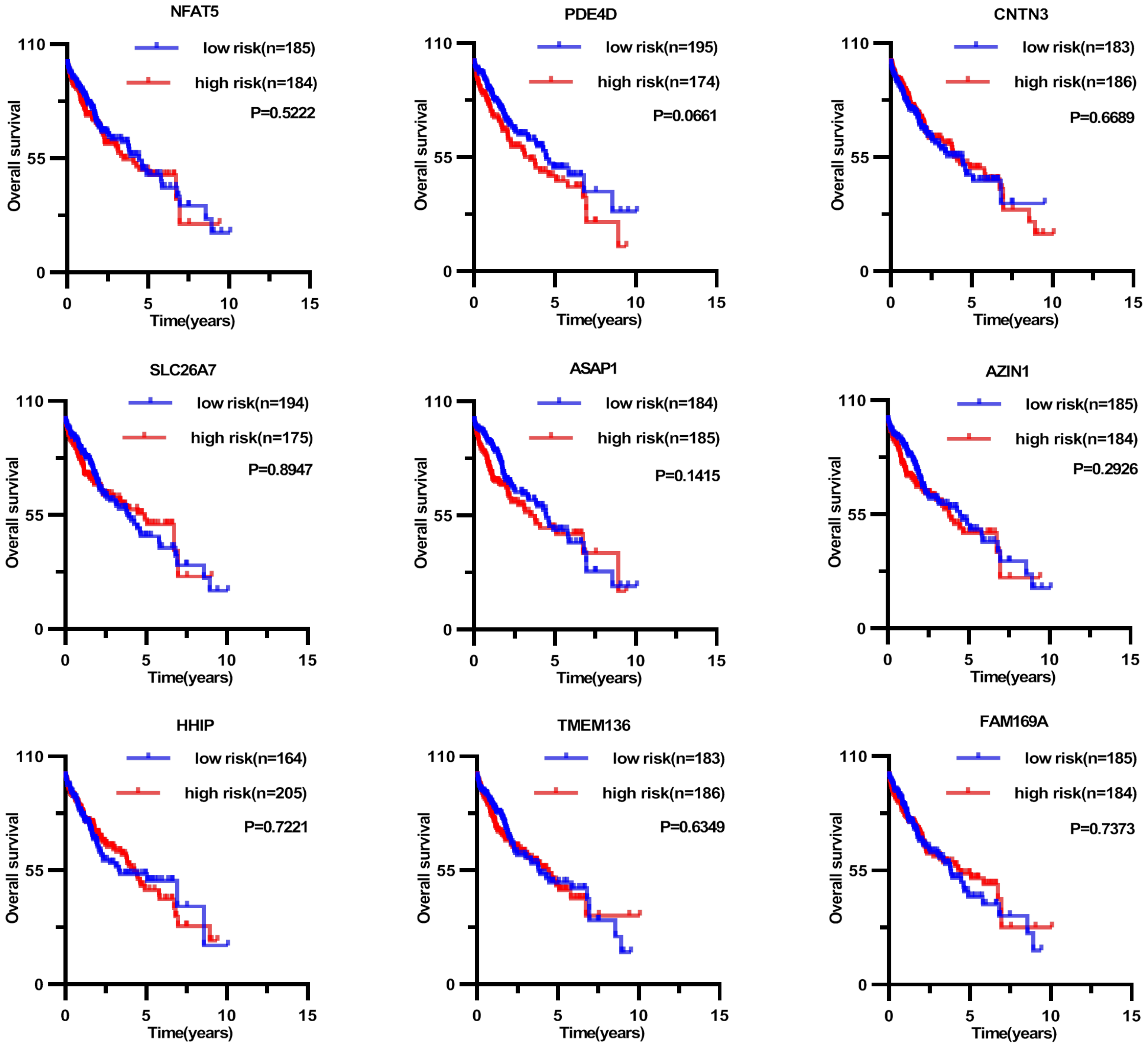

Fig. S3. Kaplan–Meier analysis of genes. Kaplan–Meier of genes that have no survival significance in the ceRNA network.

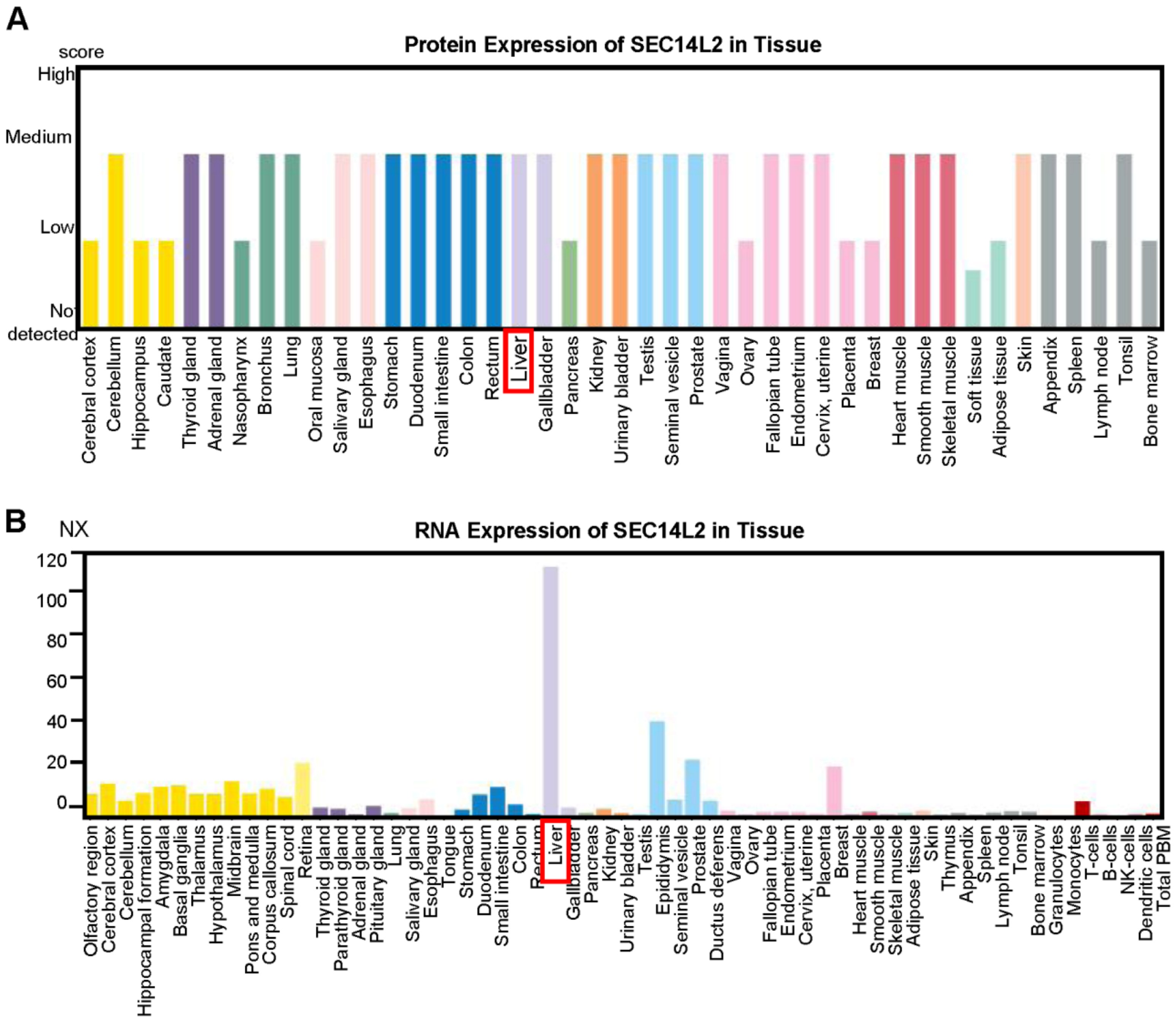

Fig. S4. The levels of SEC14L2 in various normal organ tissues. (A) Protein and (B) transcription levels of SEC14L2 in various normal organ tissues according to the HPA database.

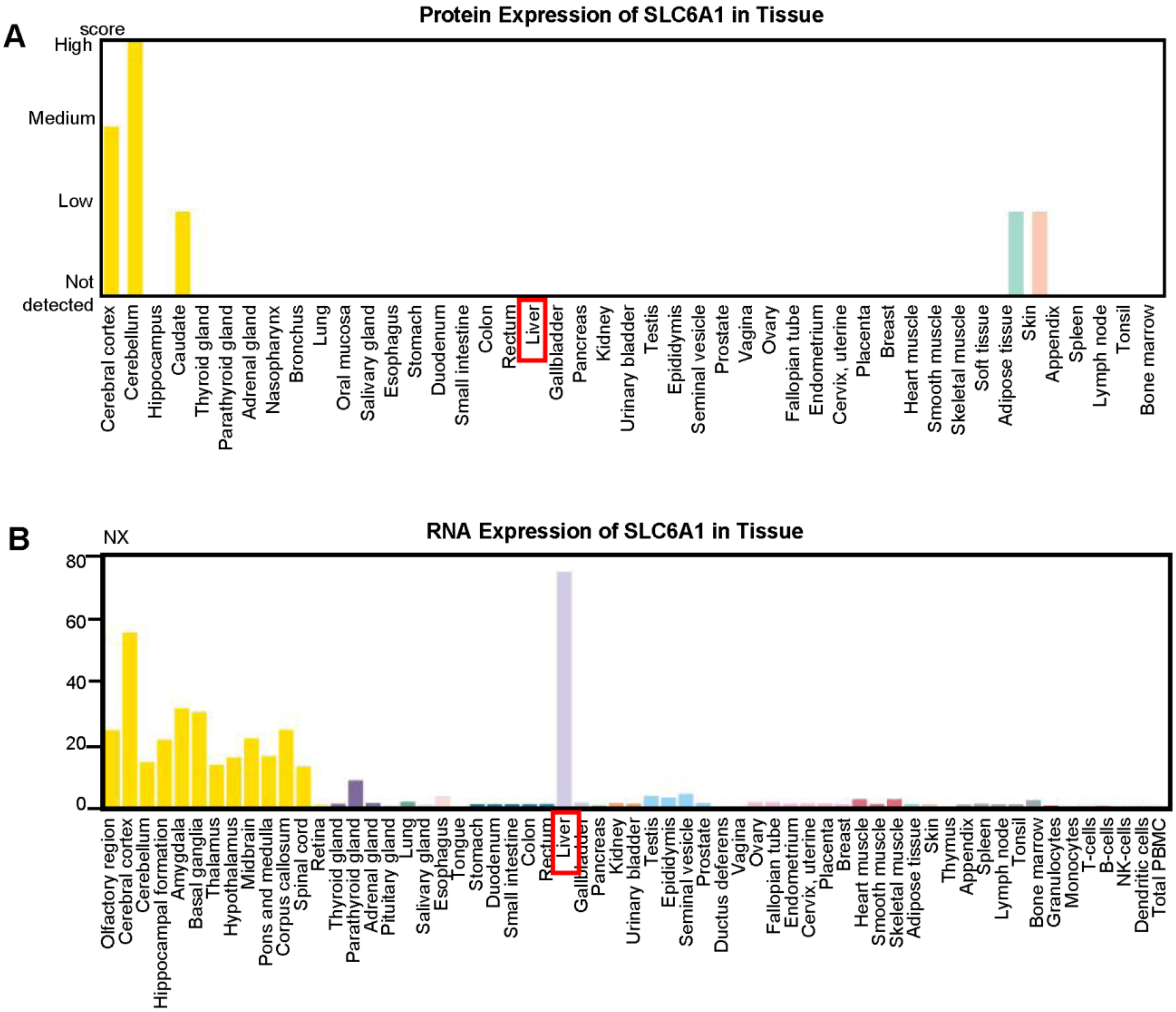

Fig. S5. The levels of SLC6A1 in various normal organ tissues. (A) Protein and (B) transcription levels of SLC6A1 in various normal organ tissues according to the HPA database.

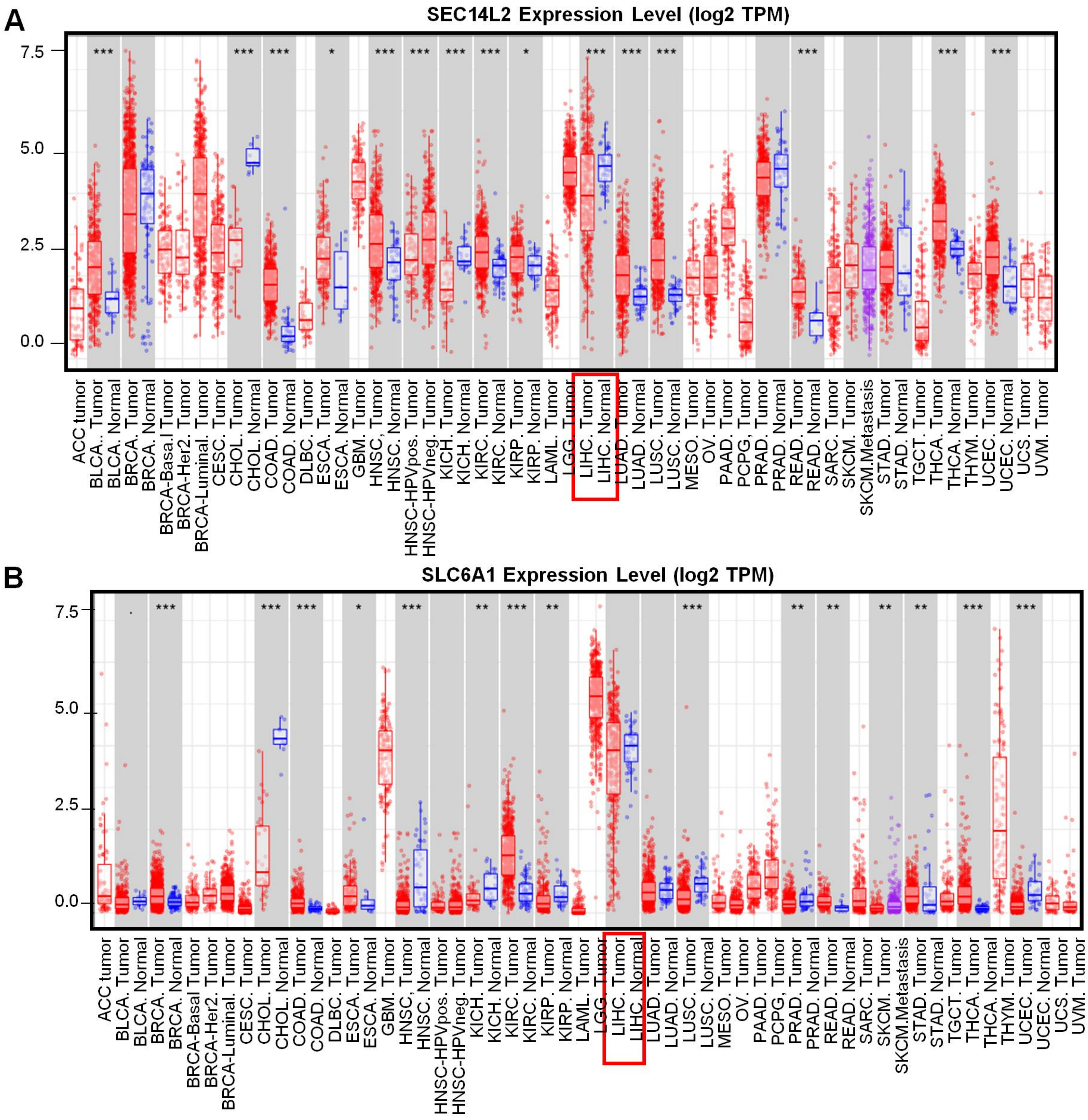

Fig. S6. The SEC14L2 and SLC6A1 expressed levels. (A) SEC14L2 and (B) SLC6A1 expression in different tumor tissues according to the TCGA database.

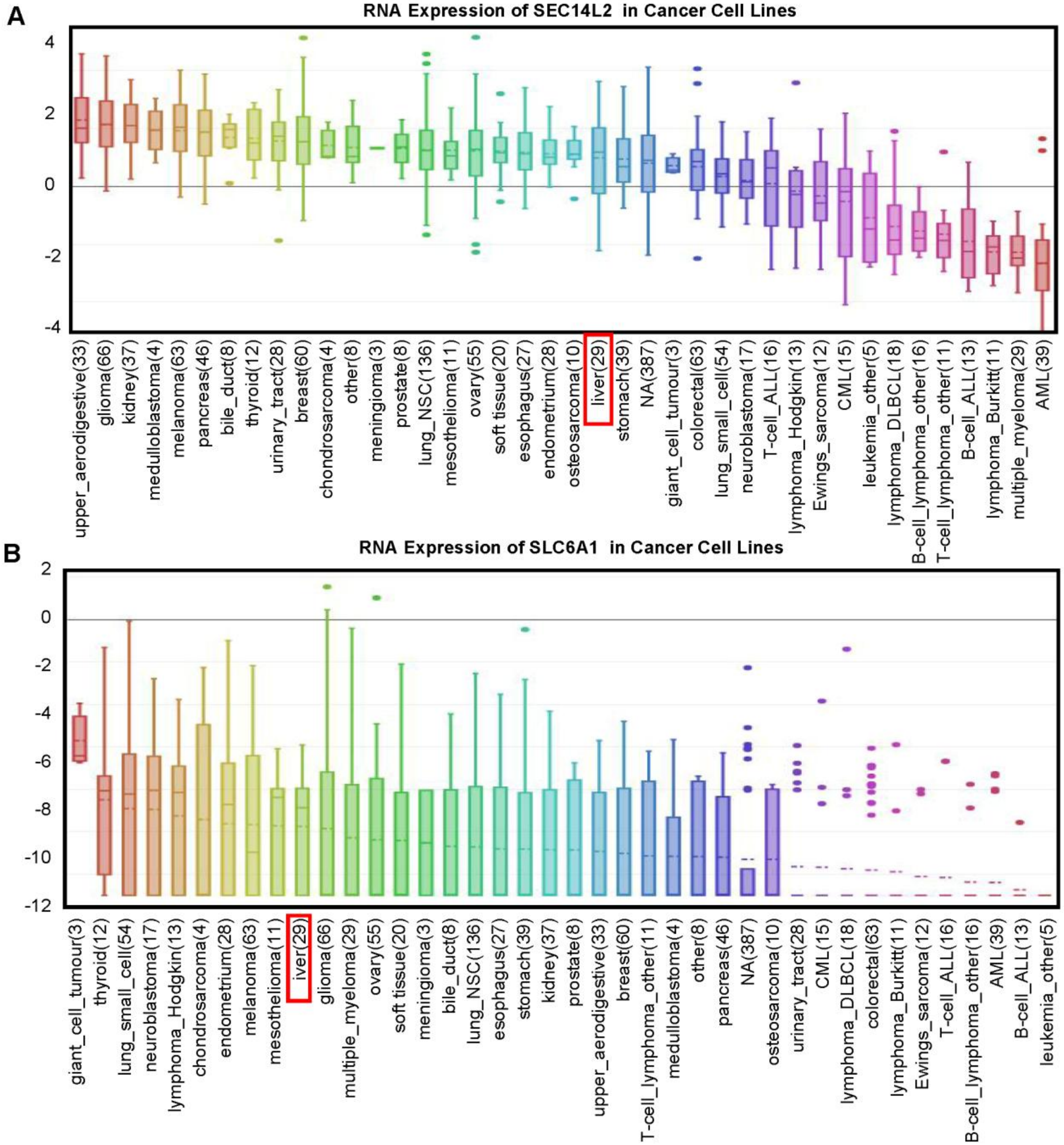

Fig. S7. SEC14L2 and SLC6A1 expression levels in cancer cell lines. The expression of (A) SEC14L2 and (B) SLC6A1 in cancer cell lines according to the Broad Institute CCLE.

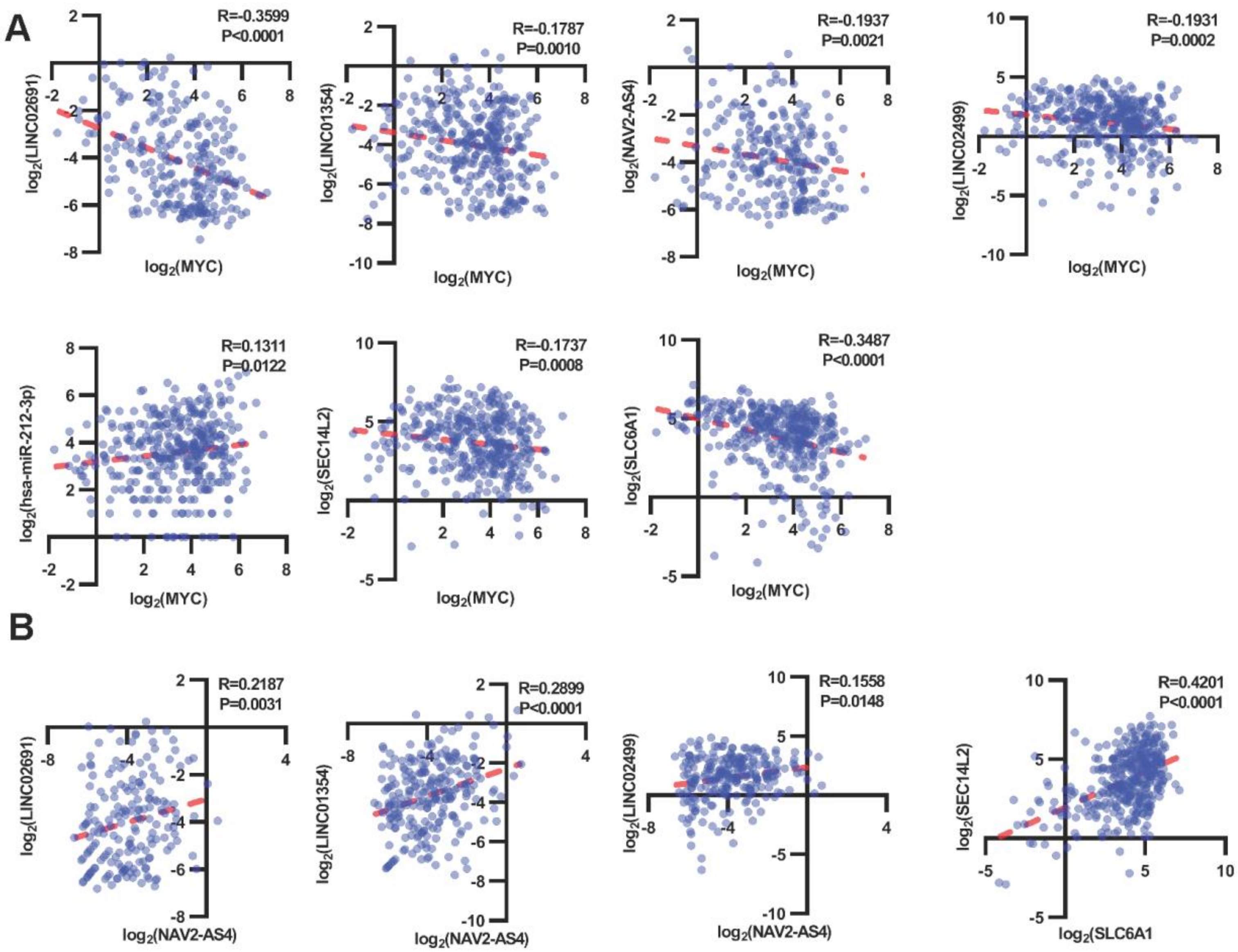

Fig. S8. Correlation among lncRNAs, miRNAs, and mRNAs. (A) Correlation between Myc and lncRNAs, miRNA, and mRNAs. (B) Remaining correlation among lncRNAs, miRNAs, and mRNAs.
